# Systematic investigation of pre-processing and feature extraction techniques in medical image analysis

**DOI:** 10.1007/s44163-026-01768-1

**Published:** 2026-07-22

**Authors:** Pegah Dehbozorgi, Oleg Ryabchykov, Thomas W. Bocklitz

**Affiliations:** 1 Leibniz Institute of Photonic Technology, Member of Leibniz Health Technologies, Member of the Leibniz Centre for Photonics in Infection Research (LPI), Albert-Einstein-Strasse 9, 07745 Jena, Germany; 2 Institute of Physical Chemistry (IPC) and Abbe Center of Photonics (ACP), Member of the Leibniz Centre for Photonics in Infection Research (LPI), Friedrich Schiller University Jena, Helmholtzweg 4, 07743 Jena, Germany

**Keywords:** Medical image processing, Binary disease detection, DL-based feature extraction, Image pre-processing, Pre-trained networks

## Abstract

As medical imagery remains the cornerstone of diagnosis, the success of complex classification tasks depends on high-quality data and diagnostic features. This study evaluates image pre-processing and feature extraction to optimize model performance. Our investigation assesses how variations in pre-processing and feature extraction impact classification efficiency across three imaging modalities: radiology (chest X-ray images), pathology (H&E (Hematoxylin and Eosin)-stained patches), and ophthalmology (OCT (Optical Coherence Tomography) scans). The experimental framework incorporates nine pre-processing techniques, adjustment (brightness, contrast, and histogram equalization), filtering (mean, median, and Gaussian), and three normalization schemes, systematically combined for each modality. Features were extracted using five pre-trained architectures (VGG16, ResNet50, DenseNet121, MobileNetV2, and InceptionV3) and classified via a PCA-LDA (Principal Component Analysis-Linear Discriminant Analysis) pipeline. Performance was evaluated using the mean sensitivity score. Mean sensitivity scores improved significantly: H&E-stained images increased from 74·9% to 96·95%, chest X-rays from 89·9% to 96·65%, and OCT scans from 82·4% to 98·8%. No single pre-processing configuration consistently dominated; optimal settings varied by modality, confirming the absence of a universal solution. VGG16 and DenseNet121 exhibited the greatest robustness. Implementing at least one pre-processing step combined with a robust DL feature extractor can substantially improve model efficacy in diagnostic detection tasks.

**Supplementary Information:**

The online version contains supplementary material available at 10.1007/s44163-026-01768-1.

## Introduction

The field of medical research and clinical diagnosis is heavily reliant on the evidence provided by image analysis [1]. Diagnostic modalities, including X-ray [2], MRI (magnetic resonance imaging) [3], and OCT [4], enable experts to visualize internal abnormalities. However, the diversity of images, disease progression, and specialist burnout can lead to misinterpretations [5]. The integration of computer-aided detection technology, driven by DL (Deep Learning) and ML (Machine Learning) has been identified as a means to address these limitations, through the enhancement of interpretation accuracy and efficiency [6–9]. From a clinical perspective, these tools can act as a robust second opinion, potentially reducing the diagnostic variability that exists between different practitioners.

ML algorithms allow systems to identify patterns with minimal intervention, while DL models utilize multi-layer neural networks to acquire intricate, hierarchical data representations [10]. These techniques reveal hidden anomalies challenging to the human eye, thereby supporting personalized care. Despite these advancements, raw medical images are often corrupted by noise, uneven illumination, blurriness, or poor contrast during acquisition. Such factors have been demonstrated to degrade image quality, thus necessitating the implementation of pre-processing techniques to ensure the diagnostic reliability of any AI (Artificial Intelligence)-based analysis pipeline [11]. In clinical practice, ensuring this reliability is essential, as the difference between a high-quality scan and a noisy one can result in life-altering false positives or overlooked pathologies.

Pre-processing is defined as the initial operations performed on raw images with the purpose of enhancing their quality and preparing them for the subsequent analysis. This crucial stage standardizes data and highlights relevant attributes by correcting lighting variations, noise, or contrast. Common techniques include adjusting brightness, applying noise-reduction filters, and employing normalization to scale pixel intensities. Depending on image complexity, researchers may apply a single method or a strategic combination of techniques. Following pre-processing, feature extraction identifies the most significant information from images. Rather than analyzing every pixel, this stage reduces the data to a compact set of descriptive attributes used as inputs for ML or DL classification models [12]. In medical imaging, features are distinct visual patterns, such as textures, shapes, and pathological structures, essential for diagnosis. The effectiveness of classification is reliant upon the interplay between pre-processing and feature extraction; well-defined features empower models to produce more accurate clinical predictions.

In medical imaging research, the choice of pre-processing and feature extraction techniques is often based on convention or prior studies rather than systematic comparison. This practice leads to arbitrary decisions and creates a clear research gap. The absence of a comprehensive study that systematically evaluates widely used methodologies for developing predictive models in medical imaging, particularly with respect to pre-processing and feature extraction, motivated this work. These components are critical for ensuring the accuracy and robustness of predictive models, yet their combined impact remains insufficiently explored. To address this gap, we investigate how different pre-processing strategies, in conjunction with various feature extraction techniques, influence the performance of medical image classification tasks. We argue that the careful integration of these stages into the model development pipeline is essential for building effective classification systems, which can ultimately contribute to both scientific understanding and real-world applications, especially in domains such as medicine, where precise classification is of paramount importance.

To the best of our knowledge, this study is the first to systematically and comprehensively analyze the combined effects of pre-processing and feature extraction on binary classification outcomes across three medical imaging modalities: radiology, pathology, and ophthalmology. Our approach is distinguished by its methodological integrity and the diversity of the input data.

Building on our previous work [13], which proved that pre-trained DL networks can substantially improve medical image analysis when applied as feature extractors, the present study employs five established DL architectures (VGG16, ResNet50, DenseNet121, MobileNetV2, and InceptionV3) for deep feature extraction. The extracted features are then reduced via PCA [14] and classified using LDA [15], with mean sensitivity (equivalent to balanced accuracy) as the primary performance metric. By systematically combining a broad range of pre-processing techniques and exploring all the possible combinations across diverse imaging modalities, we aim to identify optimal configurations, if they exist, and to demonstrate the necessity of a tailored, evidence-based approach to model development in medical imaging.

## Materials and methods

**Fig. 1:**
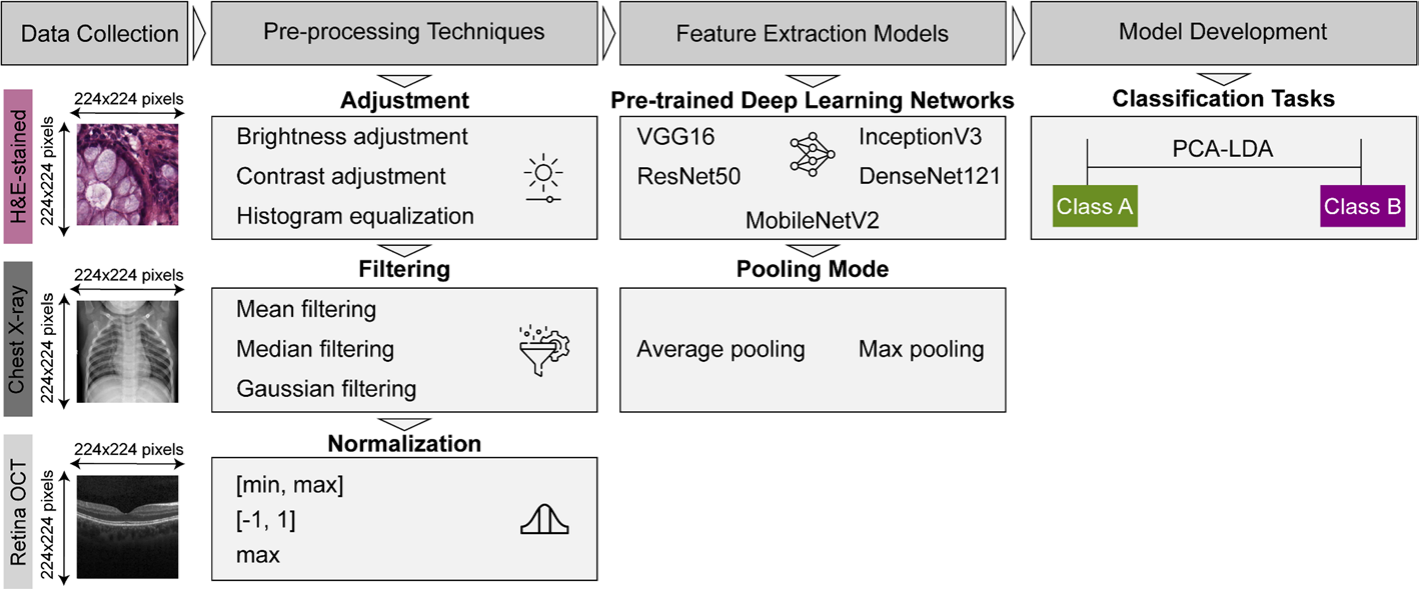
Overview of the methodological workflow. The figure illustrates the end-to-end experimental pipeline employed in this study, comprising four sequential stages. In the first stage, data collection, images were sourced from three distinct medical imaging modalities: H&E-stained histopathology, chest X-ray, and retina OCT, all standardized to a resolution of 224 × 224 pixels. In the second stage, pre-processing, each image was subjected to techniques from three groups: adjustment methods (brightness adjustment, contrast adjustment, and histogram equalization), filtering methods (mean, median, and Gaussian filtering), and normalization strategies ([min, max], [− 1, 1], and max normalization). In the third stage, feature extraction, deep features were extracted using five pre-trained networks, VGG16, ResNet50, MobileNetV2, InceptionV3, and DenseNet121, operating in either average pooling or max pooling mode. In the fourth and final stage, model development, the extracted features were used to train a PCA-LDA classifier to perform binary classification

This study follows a structured and systematic experimental workflow, schematically illustrated in Fig. 1. In summary, medical images collected from multiple imaging modalities were subjected to a series of pre-processing operations before being passed to the pre-trained networks for feature extraction. The resulting feature representations were subsequently used to train a simple linear classifier for binary classification. By combining different pre-processing strategies and feature extraction configurations, a comprehensive set of experimental pipelines was constructed and evaluated. The following subsections describe each component of this workflow in detail.

### Description of image datasets

This study used three publicly available medical image datasets to ensure generalizability and robustness in medical image analysis. We intentionally selected datasets from three distinct domains commonly used in routine clinical practice: pathology (H&E-stained tissue patches), radiology (chest X-rays), and ophthalmology (retina OCT scans). This diversity is critical because each modality relies on fundamentally different imaging physics; colorimetric staining in histology, transmission-based imaging in X-rays, and interferometric reflectance in OCT. Evaluating our methods across these varied data sources allows us to determine whether pre-processing and feature extraction strategies are universally applicable or require domain-specific adaptation. The key characteristics of the three datasets are summarized in Table 1.

**Table 1:** Summary of the integrated datasets. This table provides a detailed overview of all datasets integrated into the study.

Imaging modality	Pathology: H&E-stained dataset	Radiology: Chest X-ray dataset	Ophthalmology: Retina OCT dataset
Classes	Normal (NORMAL) vs. Tumorous (TUM)	Clear lungs (NORMAL) vs. Pneumonia (PNE)	Healthy retina (NORMAL) vs. Choroidal neovascularization (CNV)
Image dimensions	224 × 224 pixels	224 × 224 pixels	224 × 224 pixels
Training instances	10,000 (5000 per class)	10,000 (5000 per class)	10,000 (5000 per class)
Test instances	2000 (1000 per class)	2000 (1000 per class)	2000 (1000 per class)
Total instances	12,000	12,000	12,000

It specifies key characteristics of each dataset, including the imaging modality used, the target classification categories or labels, image dimensions, and the distribution of instances across the training and testing sets. The total number of samples is also reported

The original data providers obtained all necessary ethical approvals and informed consent. Because the datasets are already publicly released, no additional ethical approval or informed consent was required for this secondary analysis.

#### Hematoxylin and eosin-stained (H&E-stained) dataset

H&E staining is a widely used histological and pathological technique that enables the detailed visualization of tissue morphology by creating distinct color contrasts between cellular and structural components. Often considered the gold standard in histopathology, H&E staining plays a key role in diagnosing diseases such as cancer and is routinely used to evaluate biopsy samples [16]. In our study, we used a publicly available dataset of H&E-stained image patches extracted from human colorectal cancer (CRC) and normal tissue samples [17]. These patches were uniformly resized to 224 × 224 pixels and categorized into two classes: normal tissue (NORMAL) and colorectal adenocarcinoma epithelium tumor tissue (TUM).

#### Chest x-ray dataset

X-ray imaging uses electromagnetic radiation to produce images of the body’s internal structures. Tissues absorb X-ray photons according to their density: dense materials such as bones appear white, soft tissues appear gray, and air appears black. This technique is widely used in clinical practice to diagnose conditions such as fractures, pneumonia, and other internal abnormalities [18]. To broaden the scope of our analysis, we also included chest X-ray images from a publicly available dataset. The dataset contains normal chest X-rays with clear lungs (NORMAL) and images showing pneumonia (PNE). All images were resized to 224 × 224 pixels [19].

#### Optical coherence tomography (OCT) dataset

OCT is an imaging technique that provides high-resolution, cross-sectional views of biological tissue by analyzing the light reflected from different layers. In ophthalmology, OCT is widely used to visualize retina structures and support the diagnosis of retina diseases by providing detailed images of the layers of the retina [18]. The final modality included in this study consists of publicly available OCT images from adult patients [19]. The dataset contains normal retina scans (NORMAL) and scans showing choroidal neovascularization (CNV). All images were resized to 224 × 224 pixels.

**Fig. 2:**
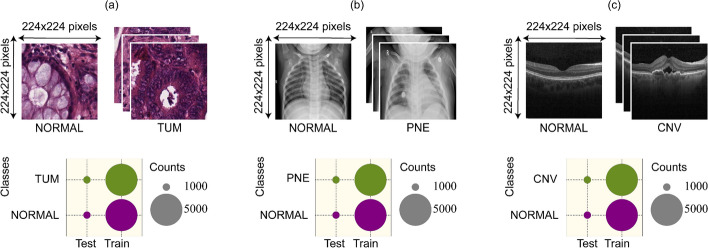
Characteristics of the analyzed images across each dataset. This figure presents an overview of the image datasets used in this study; all uniformly resized to 224 × 224 pixels to ensure consistency during model training and evaluation. **a** H&E-stained images: Representative examples of H&E-stained tissue patches are shown. Images labeled as NORMAL correspond to healthy tissue samples, whereas those labeled as TUM represent tumorous tissue regions. For both classes, 5000 images were used for training and model development, and 1000 images were allocated for testing and performance evaluation. **b** Chest X-ray images: Sample images are displayed for both classes. Images labeled as NORMAL depict healthy lungs, while those labeled as PNE correspond to lungs diagnosed with pneumonia. Each class included 5000 images for model training and 1000 images for testing and validation. **c** OCT images: Examples of OCT scans of the retina are provided. The NORMAL class represents healthy retina structure, while the CNV class includes scans showing choroidal neovascularization. Similar to the other datasets, 5000 images per class were used for training, and 1000 images per class were reserved for testing and evaluation. Overall, this figure highlights the diversity and balanced composition of the datasets across three distinct medical imaging modalities, used for the comparative model analysis

Figure 2 presents examples from the three medical image datasets used in this study. To enable a fair comparison across modalities, each class was balanced with 5000 images for training and 1000 images for independent testing. This uniform split, together with identical image dimensions, was applied consistently across all three integrated datasets.

### Image pre-processing

Raw data needs to be systematically pre-processed before it can be analyzed effectively by ML models. This initial phase involves transforming and encoding the data to ensure it is in a suitable format for efficient feature extraction and algorithmic parsing [20]. A comprehensive analytical framework usually involves applying multiple pre-processing methods sequentially to create a consistent strategy. Each method targets a specific limiting factor in an image. Selecting and ordering these techniques appropriately is critical, as an inappropriate configuration can drastically reduce a model’s predictive accuracy [21].

Current digital image enhancement techniques can generally be categorized as either spatial or frequency domain methods. Spatial domain techniques directly modify image pixels by adjusting their intensity values to achieve the desired enhancement. In contrast, frequency domain methods transform the image into the frequency domain via the Fourier transform. Then, they apply enhancement operations to the transformed data. Finally, they use the inverse Fourier transform to obtain the resultant image. These operations effectively adjust brightness, contrast, and gray level distributions, thus modifying the pixel intensities of the output image according to the applied transformation function [22].

A key goal of this research is to assess the impact of pre-processing methods on the medical image classification. To this end, common spatial domain methods were utilized to mitigate noise and enhance image quality. The pre-processing phase systematically implemented and combined nine distinct techniques, including brightness and contrast adjustment, histogram equalization, and mean, median, and Gaussian filtering. Three different normalization strategies were also incorporated, [min, max], [− 1, 1], and max scaling, along with a no-preprocessing baseline for comparison. We constructed 124 unique pre-processing combinations and evaluated each under two pooling modes (average and maximum) for feature extraction. See Sect. 2.3 for details on feature extraction. The process involved three sequential stages: adjustment, filtering, and normalization, applied in a fixed order. This resulted in 248 pipelines per dataset. The 124 combinations were derived from three cases: (A) combining three adjustment methods (brightness, contrast, and histogram equalization) with nine filtering configurations (mean, median, and Gaussian filters, each with three parameter settings), yielding 108 combinations; (B) applying the three adjustments without filtering across four normalization schemes for 12 combinations; and (C) applying no adjustments or filtering across four normalization schemes for four combinations. A complete count of all the combinations is provided in the supplementary material sect. S4.

We specifically selected the adopted adjustment and filtering techniques as they represent the most fundamental and widely cited methods for image enhancement and noise reduction in the spatial domain. These techniques are simple, effective, and applicable to almost all imaging modalities, and they form a baseline of standard procedures in many image analysis pipelines. Our pre-processing strategy, including adjustment, filtering, and normalization, is thoroughly outlined in sects. S1, S2, and S3 of the supplementary materials. Each section provides comprehensive details about the techniques employed.

As shown in [11], the order in which the pre-processing steps of adjustment, filtering, and normalization are applied has a minimal effect on the classification outcomes. Consequently, this study adopted a consistent three-stage pipeline consisting of adjustment, followed by filtering and normalization. Table 2 provides an overview of the pre-processing methods implemented in this study.

**Table 2:** Overview of the applied pre-processing techniques. This table provides a detailed overview of the data pre-processing methods employed, including the selected adjustment techniques, filtering methods, and normalization schemes used to enhance data quality

Index	3	2	1	0
Adjustment method	Brightness adjustment	Contrast adjustment	Histogram equalization	No adjustment
Filtering method	Mean filtering	Median filtering	Gaussian filtering	No filtering
Kernel size	(3 × 3)	(5 × 5)	(7 × 7)	No kernel
Sigma value	0.5	1	2	No sigma
Normalization method	[min, max]	[− 1, 1] range	(max)	No normalization

**Fig. 3:**
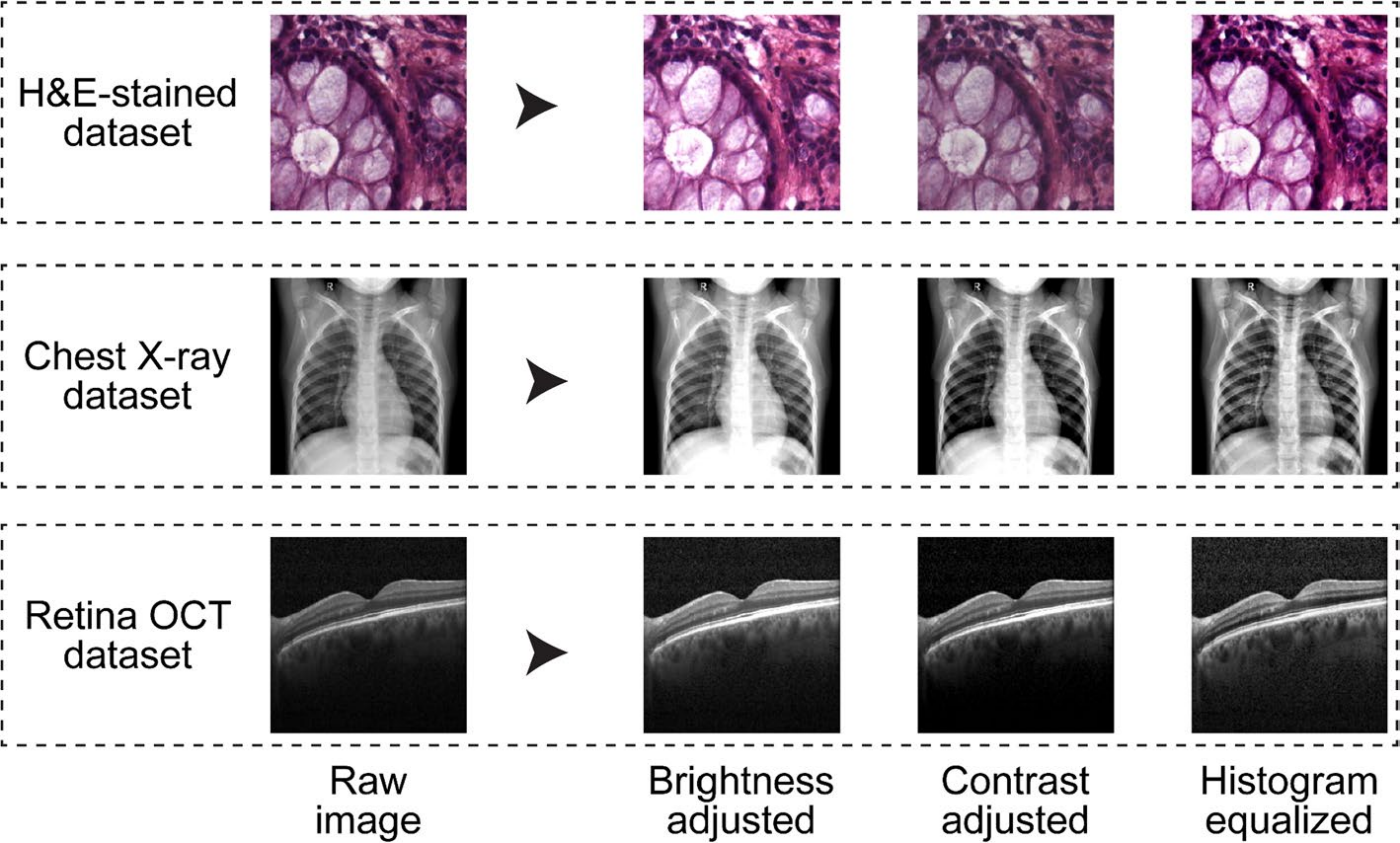
Image transformation through various adjustment techniques. This figure demonstrates how different image adjustment methods, brightness adjustment, contrast adjustment, and histogram equalization affect visual characteristics across three medical imaging modalities: H&E-stained histopathology, chest X-ray, and retina OCT images. Each technique alters the pixel intensity distribution in distinct ways: brightness adjustment shifts overall intensity levels, contrast adjustment amplifies or compresses differences between light and dark regions, and histogram equalization redistributes intensities to enhance global contrast. These transformations aim to improve the visibility of diagnostically relevant structures and highlight subtle image features that may otherwise be difficult to discern

**Fig. 4:**
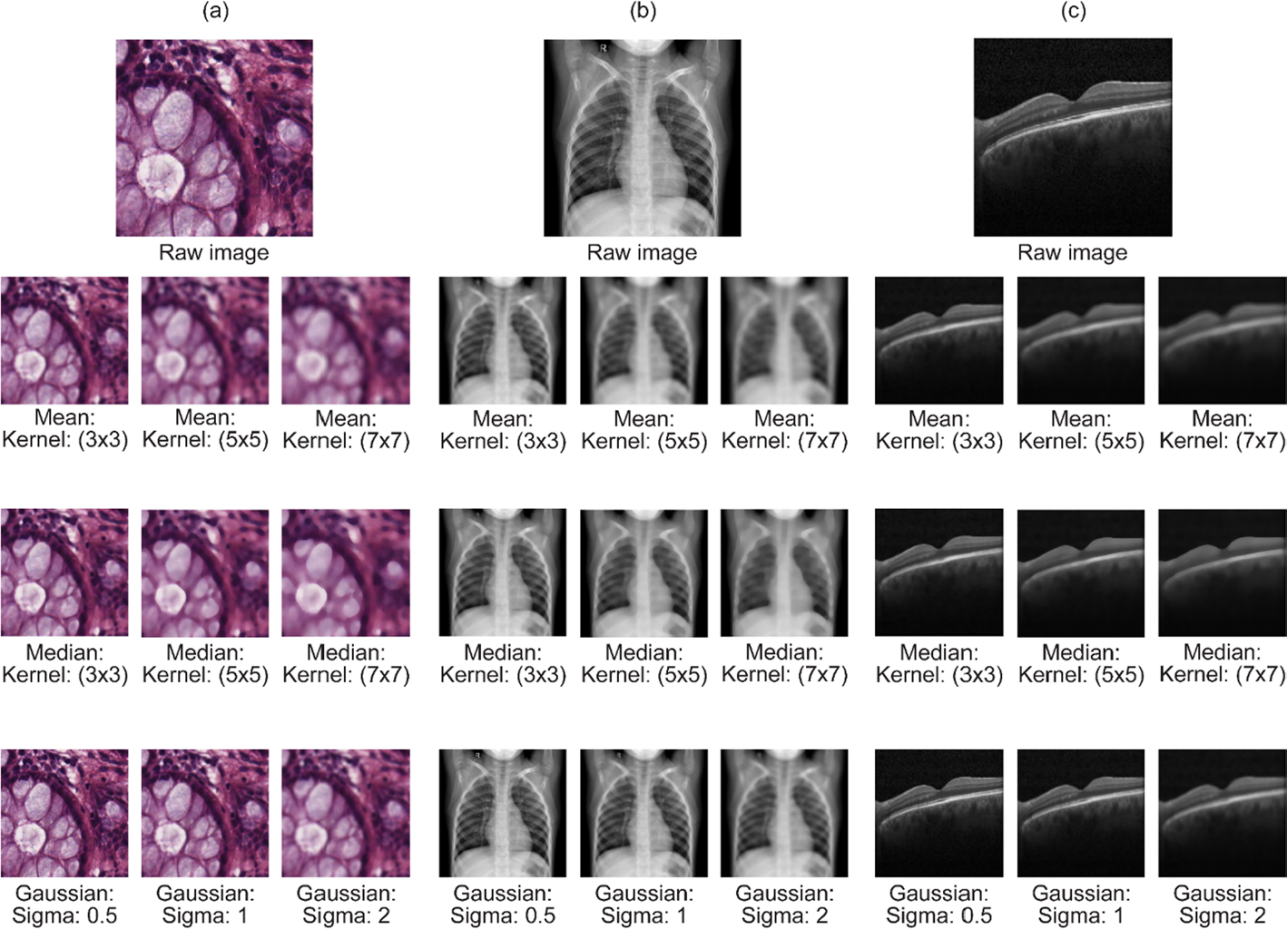
Image transformation through various filtering techniques. This figure demonstrates the effects of different spatial filtering methods, mean, median, and Gaussian filters, applied to representative samples from three distinct medical imaging datasets: **a** H&E-stained histopathology images, **b** chest X-ray images, and **c** retina OCT scans. Each filtering technique is applied using a range of kernel sizes and sigma values to illustrate the resulting changes in texture, contrast, and structural detail. These transformations aim to enhance image features and reduce background noise, thereby improving visual interpretability for subsequent analysis. However, excessive smoothing caused by large kernels or high sigma parameters can blur important diagnostic details, emphasizing the need to balance noise suppression with feature preservation when pre-processing medical images

Representative transformations are illustrated in Figs. 3 and 4. Figure 3 presents the examples of adjustment techniques, including brightness and contrast adjustments alongside histogram equalization. Figure 4 visualizes image transformations after applying filtering techniques with varying levels of effectiveness by employing different kernels and sigma values.

### Deep feature extraction

Feature extraction transforms raw data into numerical features that are machine-readable and relevant to the data. This process enables more efficient analysis by preserving the essential information from the original data. Additionally, feature extraction reduces redundant data, facilitating faster learning and generalization in the ML process. This leads to several benefits, including improved accuracy, reduced risk of overfitting, faster training, enhanced data visualization, and increased interpretability of the model [23–25]. Advanced DL approaches, particularly through the implementation of pre-trained DL models, have proven to be efficient when employed as feature extractors, offering both high accuracy and low latency [13, 26].

This strong performance record is why we have chosen to focus on DL-based approaches for feature extraction in the current study. The pre-trained DL models incorporated are listed below:

1. *VGG16* [27], is a convolutional neural network (CNN) developed by the Visual Geometry Group (VGG) at the University of Oxford. It is characterized by 13 convolutional and three fully connected layers. Its architecture employs a sequential series of convolutions and max pooling to extract hierarchical visual features, which serve as inputs for ML algorithms to generate reliable and accurate predictions.
2. *ResNet50* [28], utilizes a 50-layer architecture integrated with residual connections within identity and convolutional blocks. These connections allow the network to learn residual functions that map inputs directly into outputs, effectively mitigating the vanishing gradient problem and enabling the successful training of significantly deeper networks.
3. *DenseNet121* [29], is distinguished by its dense connectivity pattern, in which each layer receives concatenated feature maps from all preceding layers. This architecture promotes extensive feature reuse and parameter efficiency through dense blocks, resulting in a compact, highly efficient model that is inherently resistant to overfitting.
4. *InceptionV3* [30], incorporates specialized Inception modules that use parallel filters of different sizes to extract features at various scales and resolutions. This multiscale design improves the model’s ability to capture complex spatial patterns, providing a deeper understanding of diverse input data.
5. *MobileNetV2* [31], is an efficiency-oriented architecture designed for high performance in resource-constrained environments. It uses inverted residual blocks and linear bottlenecks to efficiently flow information while maintaining low-dimensional representations. This minimizes information loss and reduces computational overhead without compromising accuracy.

We selected these models to represent five influential CNN architectural patterns: sequential (VGG16), residual (ResNet50), dense (DenseNet121), Inception-based (InceptionV3), and lightweight (MobileNetV2). This variety enables us to determine which architectural families are best suited for feature extraction. These models were used exclusively as frozen feature extractors. Each network was initialized with publicly available pre-trained weights that were originally learned on the ImageNet [32] dataset. The medical images were processed using a standard forward pass to obtain feature vectors. It is important to note that the model weights remained fixed and were not updated at any stage of the experimental pipeline.

Furthermore, we deliberately excluded fine-tuning to control for confounding variables, such as hyperparameter optimization, thereby isolating the effects of pre-processing and architectural design. This approach is justified by the capacity of early-to-intermediate CNN layers to capture domain-agnostic features (e.g., edges and textures) from ImageNet that remain discriminative when transferred to highly specialized domains like medical imaging. By utilizing fixed, pre-trained weights without domain-specific refinement, we maintain a fully deterministic pipeline. This ensures that observed performance variations are directly attributable to our experimental variables.

Additionally, we examined the effect of average and max pooling on feature extraction performance by incorporating these modes in conjunction with the pre-trained DL models. Table 3 presents the spatial dimensions of high-level feature maps generated by each pre-trained model after being utilized as the feature extractor for a given image. We intentionally avoided standardizing the number of extracted features across different architectures because doing so would require adding extra layers to the networks. These layers introduce parametric transformations that could act as additional sources of variation. Including these layers would undermine our primary objective of isolating the effects of pre-processing strategies and the inherent feature extraction capabilities of each model’s original design. By not including these components, we can be sure that the experimental outcomes reflect the authentic representations learned by the pre-trained models rather than the effects of the standardization process.

**Table 3:** Number of extracted features per image using five different pre-trained DL models

Index	5	4	3	2	1
Pre-trained models	VGG16	ResNet50	DenseNet121	MobileNetV2	InceptionV3
Pooling mode	–	–	Average pooling	Max pooling	–
Feature map	(7, 7, 512)	(7, 7, 2048)	(7, 7, 1024)	(7, 7, 1280)	(5, 5, 2048)
Number of features after pooling (average/max)	512	1024	1280	2048	2048
Parameters (Millions)	138.4	25.5	8.1	3.5	23.9

In this study, we employed five widely used pre-trained DL models as the feature extractors to capture deep representations from the medical images. Each model independently processed the images, and the number of features extracted per image was recorded. These extracted features represent high-level, hierarchical patterns learned by the networks during pre-training on large datasets, which can be utilized for downstream tasks such as classification, or disease prediction. By analyzing the outputs of each network separately, we can compare their representational capacity and suitability for the specific medical imaging application.

### Model development for classification

Classification was performed using a basic PCA-LDA model as our classifier to minimize the influence of external hyperparameters during training. This approach enables us to remain focused only on the impact of different feature groups and pre-processing techniques in the model development and final evaluation. This design is intended to minimize confounding effects arising from the non-linear complexity and regularization behavior commonly associated with DL models and other ML classifiers, such as SVMs (support vector machines) or random forests. Beyond avoiding unnecessary hyperparameters, PCA-LDA was specifically chosen for its sensitivity to input feature quality: its performance is directly and transparently governed by the separability of the extracted feature space, making it an effective diagnostic tool for isolating and evaluating the contribution of pre-processing strategies and feature extraction architectures, which is the central aim of this study. Confining the classification process to this deterministic pipeline, therefore, ensures that the observed performance variations can be more reliably and distinctly attributed to the primary experimental variables under investigation.

PCA is a statistical, unsupervised technique that transforms high-dimensional data into a lower-dimensional representation while retaining the most significant information. Features extracted from the final convolutional layer of pre-trained DL models are often high-dimensional. PCA efficiently reduces this dimensionality while retaining the maximum variance. This makes subsequent LDA modeling more computationally feasible and robust against overfitting. LDA is a dimensionality reduction technique that is widely used in supervised classification problems. It models distinctions between groups by projecting features from a high-dimensional space onto a lower-dimensional one. As a supervised learning algorithm, LDA identifies a linear combination of features that best separates the classes within a dataset. This property provides a clear, interpretable measure of how well the classes can be separated after applying a specific feature extraction model or a given pre-processing step.

Combining PCA and LDA allows for the use of PCA’s dimensionality reduction capabilities and LDA’s discriminative power. We conducted an extensive analysis of PCA components, systematically evaluating configurations ranging from one to 100. Through the iterative training and testing of the PCA-LDA model with various numbers of PCA components, we identified the optimal configuration that produced the highest mean sensitivity for the independent test set. We used the maximum mean sensitivity value obtained from this analysis as a benchmark to compare the effectiveness of various pre-processing and feature extraction techniques. All computations were implemented in Python 3.9 on an AMD Ryzen Threadripper 3960X workstation with NVIDIA GeForce RTX 3090 GPU acceleration (see Software and computational analysis for hardware details).

### Software and computational analysis

The computations were carried out on a commercially available PC featuring an AMD Ryzen Threadripper 3960X 24-Core Processor (48 threads, 3.79 GHz, 128 GB RAM). An NVIDIA GeForce RTX 3090 GPU was also employed, which includes 10,496 CUDA cores, a base clock speed of 1440 MHz, and a boost clock speed of 1710 MHz. The GPU is equipped with 24,576 MB of GDDR6 memory, offering a memory bandwidth of 936.10 GB/s, a 384-bit memory interface, and a memory data rate of 19.50 Gbps. The analysis was performed using custom-written functions in Python programming language.

The computational complexity of our analytical pipeline can be evaluated across three main stages. First, we performed image pre-processing, involving spatial domain methods that operated directly on the image pixels. This stage was characterized by a relatively low computational cost. We identified feature extraction as the most computationally intensive stage of the pipeline. For each image, we conducted a forward pass through pre-trained DL networks to extract key features. The computational complexity of this step depended heavily on the network architecture, particularly its depth and the number of parameters (see Table 3 for details). For example, VGG16, with 138.4 million parameters, was significantly more demanding than MobileNetV2, which had only 3.5 million parameters. The total feature extraction workload was substantial across the full experimental set of 3720 pipelines (1240 per dataset), and execution would not have been feasible within a reasonable timeframe without GPU support. Nevertheless, using the frozen pre-trained weights ensured that each forward pass remained fully deterministic and computationally bounded, with no backpropagation or parameter updates at any stage. Finally, we performed classification using a PCA-LDA classifier, which was substantially less computationally demanding than feature extraction. The complexity at this stage was influenced primarily by the number of samples and the dimensionality of the feature vectors produced in the previous step. The PCA component scanning process (evaluating one to one hundred components per pipeline) introduced modest iterative costs, but these were negligible compared to the costs of deep feature extraction. This confirmed that the PCA-LDA stage did not constitute a computational bottleneck in the overall pipeline. In the supplementary materials, Tables S4, S5, and S6 summarize the computational cost for each dataset, including feature extraction time and memory usage based on raw, unprocessed images.

## Results

### Configuration tuning and optimization

In this section, we aim to address the following research question: To what extent does the integration of different pre-processing strategies with pre-trained DL feature extractors improve diagnostic sensitivity across diverse medical imaging modalities?

**Fig. 5:**
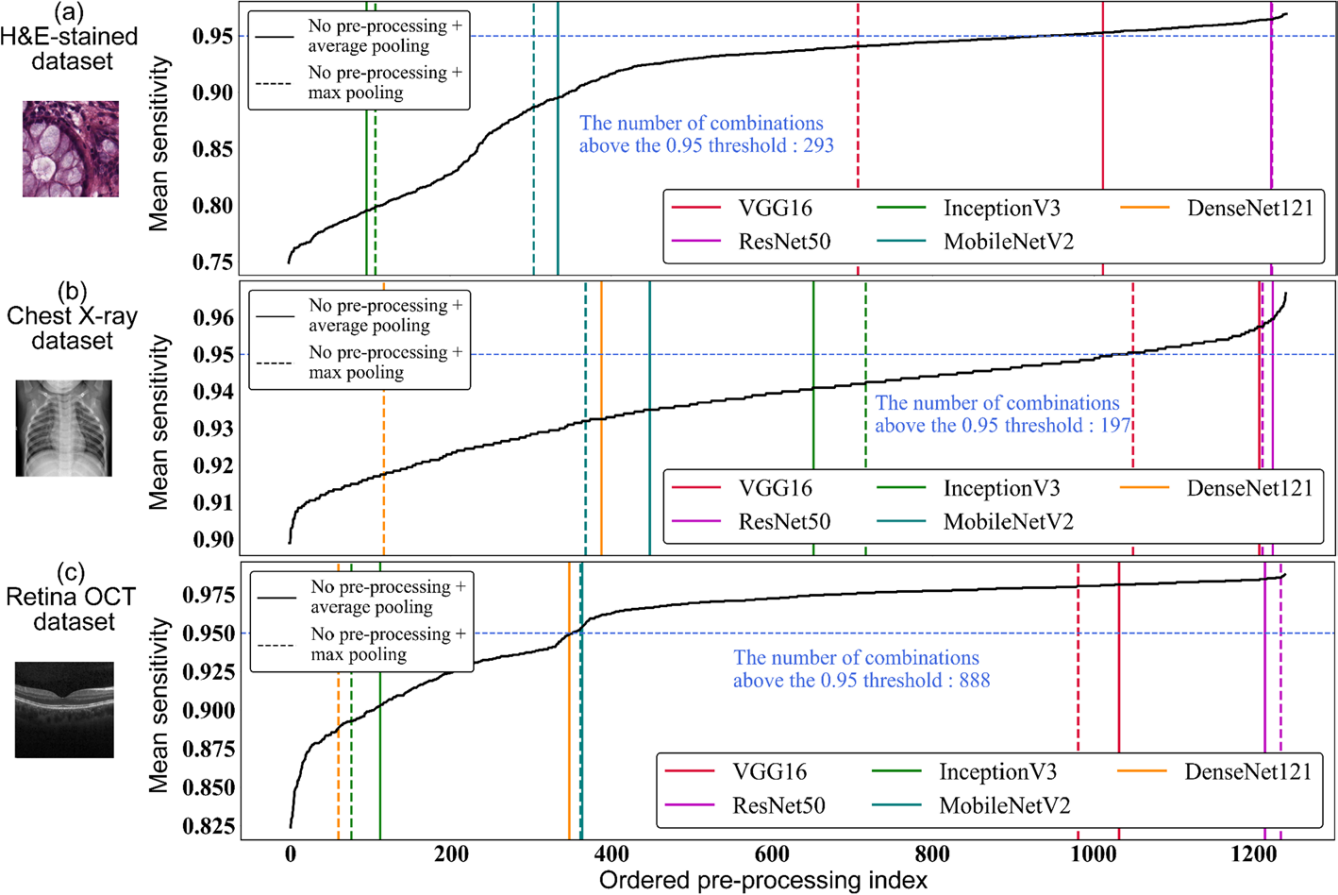
Impact of pre-processing and feature extractor optimization on the classification performance. This figure illustrates the enhancement of mean sensitivity scores across three distinct medical imaging datasets, achieved through the systematic optimization of pre-processing techniques and various DL feature extractors. Notably, both average and max pooling modes were applied universally across all pre-trained networks incorporated for feature extraction. **a** H&E-stained images: Mean sensitivity increased markedly from 74·9% to 96·95%. This improvement was primarily driven by histogram equalization and Gaussian filtering (σ = 1), applied without normalization. The ResNet50 model with average pooling was employed as the feature extractor. In contrast, the combination of brightness adjustment and median filtering (5 × 5 kernel) led to a notable decrease in the mean sensitivity, highlighting that inappropriate pre-processing can degrade the classification performance. **b** Chest X-ray images: Mean sensitivity improved from 89·9% to 96·65%. Initially, a combination of histogram equalization, median filtering (7 × 7 kernel), and normalization to [− 1, 1] with the InceptionV3 model and max pooling yielded a sensitivity of 89·9%. Subsequent optimization, including contrast adjustment, Gaussian filtering (σ = 1), and skipping of the normalization, coupled with ResNet50 and average pooling, resulted in a significant increase to 96·65%. **c** Retina OCT images: Optimization raised mean sensitivity from 82·4% to 98·8%. Initially, contrast adjustment with median filtering (7 × 7 kernel) produced lower sensitivity. After optimization, using brightness adjustment without filtering and [min, max] normalization, combined with MobileNetV2 in max pooling mode, the mean sensitivity reached its peak at 98·8%. The plots demonstrate that skipping the pre-processing phase (solid vs. dashed lines) can reduce the classification performance, highlighting the essential role of thoughtfully designed pre-processing and feature extraction strategies. Across datasets, optimization yielded 293 high-performing models for the H&E-stained dataset, 197 models for the chest X-ray dataset, and 888 models for the retina OCT dataset, all surpassing the defined performance thresholds and underscoring the effectiveness of these optimized strategies in enhancing diagnostic accuracy and potential real-world applications

Integrating pre-processing with DL feature extraction, employing both average and max pooling across all implemented pre-trained networks, yielded substantial, statistically significant gains in the mean sensitivity scores across the three modalities. For the H&E-stained dataset, the performance increased from 74·9% (worst configuration) to 96·95% (best configuration). In the context of the H&E-stained dataset (Fig. 5a), this significant improvement is primarily due to the effective use of histogram equalization during the adjustment phase and Gaussian filtering with a sigma value of 1 for noise reduction. This remarkable outcome was achieved without normalizing the H&E-stained images and using ResNet50 as the feature extractor. On the other hand, the most significant decline in the performance occurred when brightness adjustment was paired with median filtering using a 5 × 5 kernel size. Next, the images were normalized to the maximum and processed by the ResNet50 model in max pooling mode to extract features. This combination substantially reduced the mean sensitivity score, highlighting the importance of selecting pre-processing techniques compatible with the dataset’s characteristics and the model’s architecture.

A similar trend was observed in the analysis of chest X-ray images, as depicted in Fig. 5b. The performance of the classification models showed a considerable improvement, rising from 89·9% to 96·65%, depending on the pre-processing techniques and feature extraction methods employed. Initially, a combination of histogram equalization, median filtering with a kernel size of 7 × 7, and normalization to the range of [− 1, 1] was used, along with the InceptionV3 model in max pooling mode for feature extraction. This approach achieved an accuracy of 89.9%. However, by adjusting the contrast, applying Gaussian filtering with a sigma value of 1, skipping the normalization (no normalization), and utilizing the ResNet50 model with average pooling for feature extraction, the model’s accuracy significantly improved to 96·65%.

In the retina OCT images (Fig. 5c), there was a significant improvement in the mean sensitivity values, which increased from 82·4 to 98·8% after optimizing the implemented methods. Initially, the application of contrast adjustment combined with median filtering using a 7 × 7 kernel, without normalization, and the use of InceptionV3 in max pooling mode, resulted in the lowest mean sensitivity value. However, after optimizing the approach, specifically by applying brightness adjustment without filtering and normalizing the images to the [min, max] range, while employing MobileNetV2 in max pooling mode, the model achieved a remarkable increase in the mean sensitivity score, reaching a peak mean value of 98·8%.

The reported values represent the global minimum and maximum across the full set of 1240 configurations tested per dataset (248 pre-processing combinations × five different DL feature extractors). Despite the substantial differences observed in the mean sensitivity across the three investigated datasets, which already suggested a low likelihood of chance occurrence, statistical validation was performed to confirm these findings. Accordingly, the Wilcoxon signed-rank test [33] (*p* < 0·05) was applied to the results of the 1240 evaluated models per dataset, comparing the highest- and lowest-performing configurations across each modality. The analysis demonstrated that the optimized configurations significantly outperformed the lowest-performing configuration in all the cases, with highly significant p-values for the H&E-stained dataset (*p* = 1.94 × ${10}^{-18}$, 95% CI [0.2285, 0.2335]), chest X-ray images (*p* = 2.00 × ${10}^{-18}$, 95% CI [0.0647, 0.0670]), and retina OCT scans (*p* = 1.94 × ${10}^{-18}$, 95% CI [0.1703, 0.1820]). These results confirm that the observed improvements in the mean sensitivity are statistically significant and reflect the genuine gains in the classifier performance rather than random fluctuations. To enhance our analysis, we set a mean sensitivity threshold of 95%. Classification models that exceeded this benchmark were considered highly suitable for diagnostic tasks, demonstrating strong class-discrimination performance. Remarkably, for (a) the H&E-stained dataset, (b) the chest X-ray dataset, and (c) the retina OCT dataset, 293, 197, and 888 models, respectively, were identified as suitable for potential diagnostic applications through fine-tuning, pre-processing, and feature extraction procedures.

### Effect of image pre-processing

This section addresses a key question: Does a specific pre-processing method or combination of methods consistently lead to superior model performance, as measured by the mean sensitivity? For each dataset, a comprehensive analysis of 248 different combinations of pre-processing techniques has been conducted when employing a specific pre-trained DL model as the feature extractor. This extensive exploration resulted in the development of 248 distinct classification models, each reflecting a unique combination of adjustments, normalizations, and filtering methods with various kernel sizes, and sigma values. However, no single pre-processing method or combination outperformed the others consistently across the evaluated configurations.

**Fig. 6:**
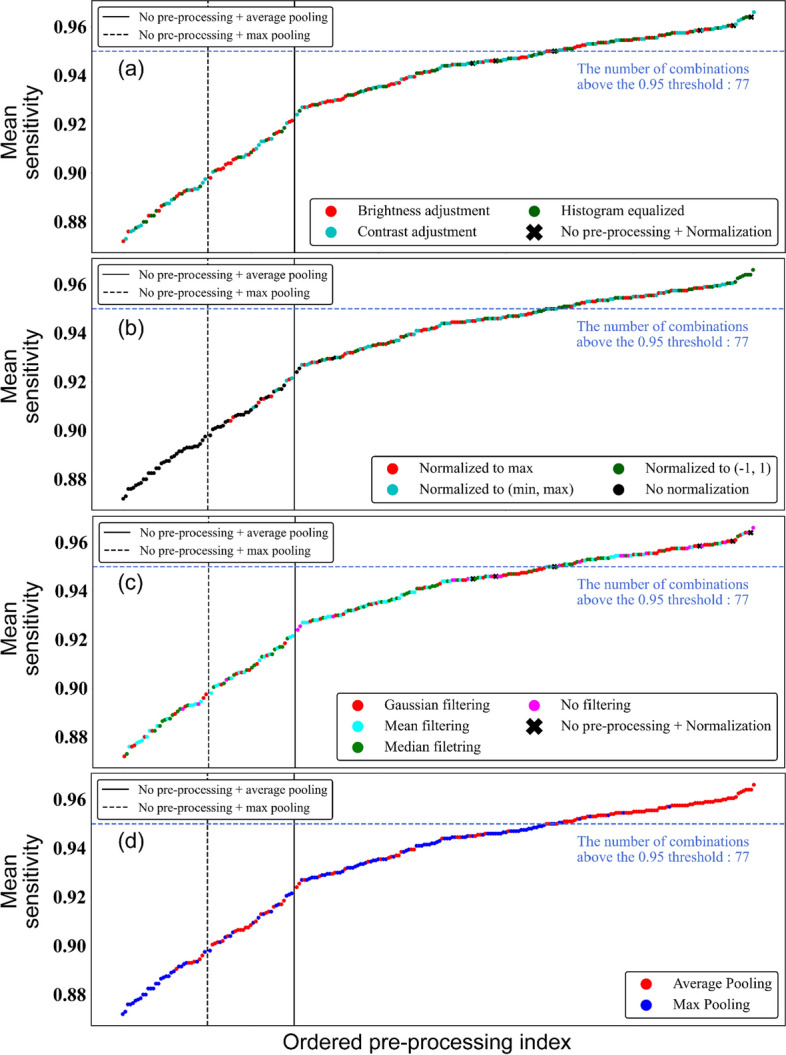
Optimization of the classification models for the H&E-stained dataset using DenseNet121 as the feature extractor. This figure presents a comprehensive evaluation of 248 distinct models, examining how various pre-processing strategies impact the binary classification performance. The analysis focuses on four key aspects: image adjustment methods, filtering techniques, normalization ranges, and pooling modes. **a** Adjustment methods: The figure shows that no single adjustment technique consistently yields higher mean sensitivity values, as evidenced by the absence of a dominant color pattern. This indicates that relying on one adjustment method alone may not be sufficient, and a combination of approaches could be necessary to maximize the model performance. **b** Normalization ranges: Comparative results for the different normalization ranges reveal that while no specific range universally improves the model performance, models trained without normalization generally exhibit lower mean sensitivity. This suggests that applying some form of normalization is beneficial in most cases, though the optimal range may depend on dataset-specific characteristics. **c** Filtering techniques: The analysis of filters with varying kernel sizes and sigma values demonstrates no uniform color pattern, emphasizing that there is no single filtering approach suitable for all scenarios. **d** Pooling strategies: The figure also compares average and max pooling for feature extraction. Average pooling consistently outperforms max pooling, indicating its superior ability to capture relevant features for the classifier and enhance the overall model effectiveness. Across all subplots (**a**–**d**), dashed blue lines indicate an established performance threshold of approximately 95% mean sensitivity. Notably, 77 models surpassed this threshold, demonstrating the potential of these optimized approaches for high-performance classification in medical imaging and diagnostic applications

Figure 6 illustrates this variability for the H&E-stained dataset using DenseNet121 as the feature extractor. Notable improvements in the mean sensitivity values were observed through experimentation with various pre-processing configurations. For example, a mean sensitivity of 87·2% was achieved by extracting features with DenseNet121 in max pooling mode, while brightness adjustment and Gaussian filtering (sigma = 2) were applied without normalization. By refining the pre-processing steps, applying contrast adjustment without filtering and normalizing the data within the range of [− 1, 1], the mean sensitivity was further increased to 96·6%.

Generally, a uniform color indicates a clear influence of the method, whereas a mixture of colors suggests an inconsistent or uncertain effect. As illustrated by Fig. 6b, normalization had the strongest and most consistent positive effect. However, this figure also reveals that results are not consistently enhanced by a single normalization range or method. The black circles imply that skipping normalization can result in significantly lower mean sensitivity values, thereby emphasizing its importance in the analysis pipeline. Adjustment and filtering choices showed no dominant pattern (see Fig. 6a and c). Interestingly, Fig. 6d reveals that applying average pooling for feature extraction significantly improved the classifier performance, yielding higher mean sensitivity values. Considering the threshold of 95%, notably, 77 classification models achieved mean sensitivity values above this level, demonstrating a strong ability to distinguish between classes with high precision. The success of so many models in meeting or surpassing the threshold suggests that carefully selecting and tuning pre-processing techniques is crucial for improving the diagnostic performance of ML models in medical imaging. As with the H&E-stained dataset, no single pre-processing method or combination of methods emerged as a universal solution for the enhanced analysis of the chest X-ray (Fig. S1 in the supplementary materials) and retina OCT (Fig. S2 in the supplementary materials) datasets. Detailed explanations are provided in the supplementary materials sect. S5.

Although we did not identify a single, universal pre-processing pipeline applicable across all studied medical imaging datasets, namely H&E-stained histological images, chest X-rays, and retinal OCT scans, our experiments indicate that normalization of pixel intensity values has the greatest impact on model performance. Among various combinations of adjustment techniques, filtering methods, and normalization strategies, normalization had the greatest impact on the final outcomes. Despite the fact that no single normalization range or technique guaranteed superior accuracy in every case, its importance was evident, especially when model performance decreased due to skipping the normalization phase. This is especially critical when working with CNNs, which are highly sensitive to the statistical properties of their input data. These models are usually trained using datasets with clearly defined initial data distributions and scales. Without proper scaling, variations in intensity can disproportionately influence feature maps, causing the network to misinterpret relevant patterns or overemphasize noise. In contrast, well-normalized inputs ensure that all features contribute proportionately, thereby improving model stability and generalization. Therefore, even though filtering and image-specific adjustments should be adapted to the characteristics of each dataset, normalization is an important step to incorporate into the image analysis pipeline.

Additionally, the supplementary materials (Tables S1, S2 and S3) provide a summary of the classification results for four key configurations using DenseNet121 as the feature extractor across all three datasets. These configurations include the optimal and suboptimal cases, which produced the highest and lowest scores, respectively, based on multiple performance metrics such as sensitivity, accuracy, specificity, and F1 score. Furthermore, two baseline scenarios were evaluated in which key features were extracted using DenseNet121 with max and average pooling, respectively, but with no pre-processing applied.

While the previous analysis focused on the impact of pre-processing strategies, this study did not limit itself to a single DL architecture as the feature extractor. The following subsection explores this comparative perspective on different feature extractors.

### Performance of different feature extractors

**Fig. 7:**
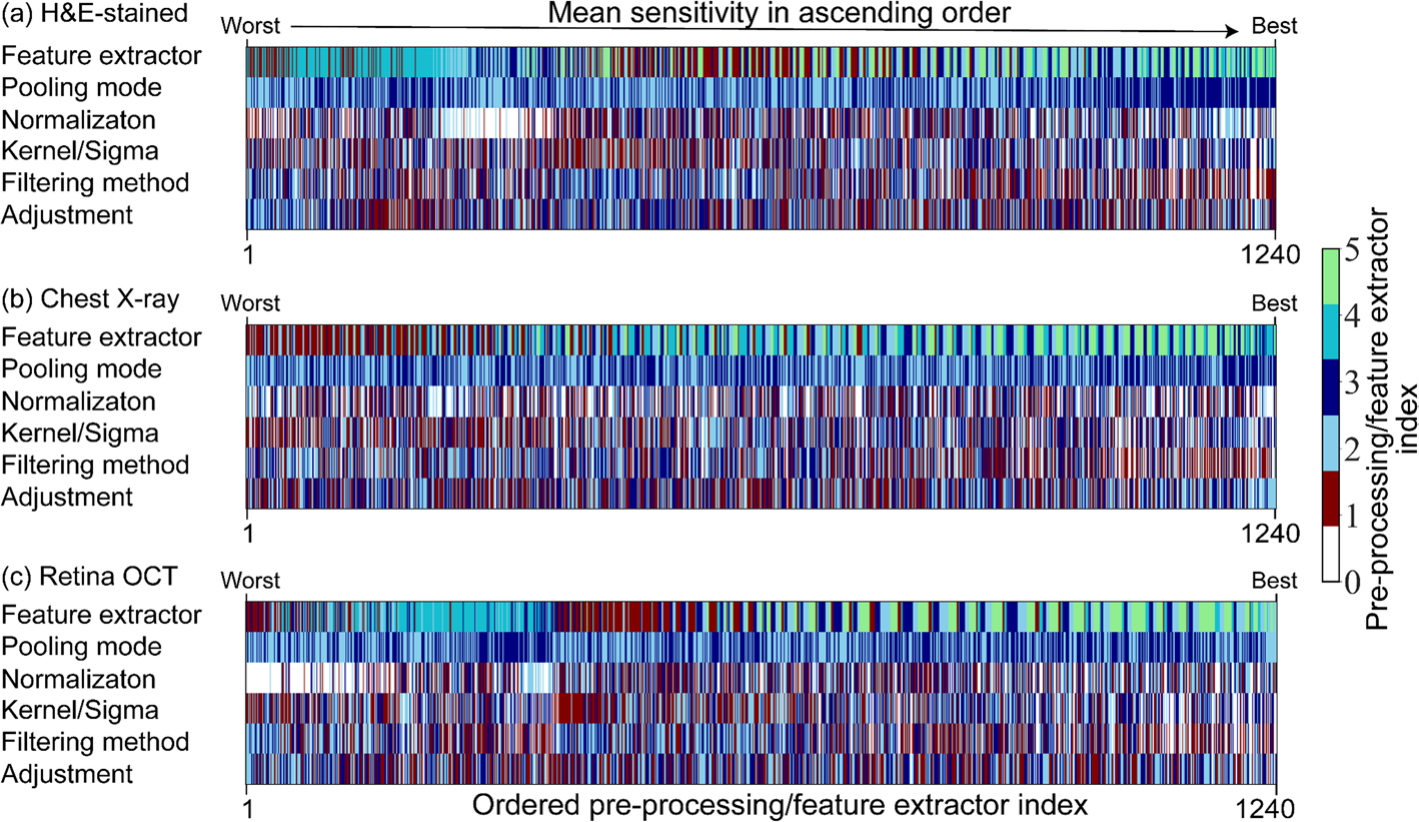
False color plot visualization, illustrating the combined effects of the pre-processing techniques and feature extraction methods on the medical image analysis. Each plot represents one of the 1240 distinct classification models, with each model defined by a unique combination of the pre-processing steps (adjustment, filtering method, kernel/sigma selection, normalization, and pooling mode) and feature extractors. Panels **a** H&E-stained images, **b** chest X-rays, and **c** retina OCT datasets illustrate that the absence of a consistent color pattern across the pre-processing methods indicates no single approach consistently outperforms others across all datasets. During feature extraction, diverse color combinations emerge, highlighting subtle differences between the models. Evaluation results show that VGG16 and DenseNet121 feature extractors achieved higher mean sensitivity, indicating improved detection performance, whereas InceptionV3 and ResNet50 exhibited lower predictive accuracy, demonstrating that the choice of feature extractor significantly influences outcomes (indexing details are provided in Table 3)

This section’s core research question is: How do different pre-trained DL feature extraction architectures, in combination with various pre-processing pipelines, influence the classification performance across multi-modal medical imaging datasets? Is there a model that performs best among the five pre-trained networks considered? Thus, to evaluate the relative contribution of the five pre-trained DL architectures, Fig. 7 presents a color-coded overview of all 1240 classification pipelines across the three datasets: (a) H&E-stained images, (b) chest X-rays, and (c) retina OCT scans. Each row corresponds to one experimental factor, including adjustment, filtering method with kernel/sigma settings, normalization, pooling mode, and feature extractor. Although pre-processing steps again showed no consistent dominant pattern, clear differences emerged among the feature extractors.

**Fig. 8:**
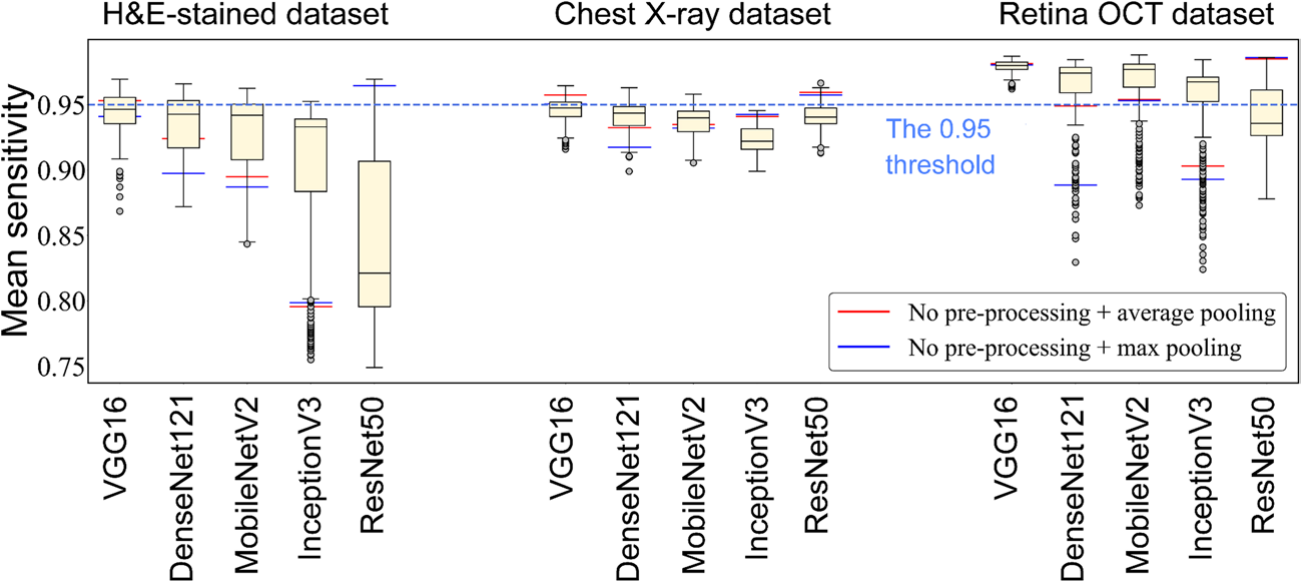
Performance comparison of the pre-trained DL feature extractors. This boxplot presents a detailed comparative analysis of several widely used pre-trained DL models (VGG16, ResNet50, InceptionV3, DenseNet121, and MobileNetV2) when employed as the feature extractors, evaluated based on the delivered mean sensitivity across multiple datasets. Mean sensitivity, reflecting the model’s ability to correctly identify positive cases, serves as a key metric for assessing feature extraction effectiveness. From the results, VGG16 consistently demonstrates superior performance, achieving both the highest mean sensitivity and low variability across all datasets, indicating robustness and reliability. DenseNet121 and MobileNetV2 also perform well, maintaining median sensitivity values at or above 95%, highlighting their strong generalization capabilities. In contrast, ResNet50 exhibits greater variability, particularly when applied to the H&E-stained dataset, suggesting sensitivity to dataset characteristics and reduced stability. InceptionV3, while more stable than ResNet50, still underperforms relative to VGG16, DenseNet121, and MobileNetV2, as indicated by the lower median values of the delivered mean sensitivity across the evaluated datasets. Overall, this analysis underscores that choice of pre-trained DL feature extractor has a significant impact on the classification performance, with VGG16 emerging as the most reliable model, followed closely by DenseNet121 and MobileNetV2

Based on the indexing in Table 3 and the color-coded visualization in Fig. 7, VGG16 and DenseNet121 produced the strongest and most robust performance across all modalities, with the majority of their pipelines yielding high mean sensitivity. In contrast, ResNet50 and InceptionV3 consistently delivered the lowest scores, confirming their comparatively limited effectiveness when used as the feature extractors in this multi-modal setting. These trends are further summarized in the comparative boxplot of Fig. 8. It provides a clearer illustration for comparing the performance of each feature extraction model across the three investigated datasets. It clearly displays the consistent superior performance of VGG16, DenseNet121, and MobileNetV2 when used as the feature extractors, as evidenced by their higher median sensitivity values. In contrast, InceptionV3 and ResNet50 exhibited relatively lower performance, with reduced median sensitivities and greater variability, indicating limited effectiveness.

In order to conduct a more in-depth investigation, the H&E-stained dataset was analyzed to evaluate the efficacy of alternative classification models, specifically PCA-SVM, PCA-RF (Random Forest), and PCA-KNN (K-Nearest Neighbors), as replacements for the standard PCA-LDA approach. The resulting classification outputs are presented as false-color maps and boxplots in Figs. S3 and S4, respectively. This comparative analysis supports our preliminary findings, as observed with the PCA-LDA; despite minor variations in the delivered mean sensitivity values among the models, the overall trends remained consistent. These findings once again demonstrated that a universal pre-processing framework remained unattainable, and that consistency within the feature extraction model is only partially preserved.

An important takeaway from Fig. 8 is that the lack of a clear pre-processing trend suggests that random combinations of pre-processing techniques paired with a stable model can produce a more robust classifier than with a less stable one. For instance, when working with H&E-stained images, using any pre-processing method with VGG16 tends to produce a more consistent and reliable classifier than using ResNet50. While ResNet50 may achieve higher sensitivity with certain pre-processing setups, VGG16 generally provides more consistent performance. This highlights the complex yet critical interplay between pre-processing strategies and feature extractors in optimizing model outcomes.

## Discussion

This study provides the first systematic, multi-modal evaluation of image pre-processing techniques combined with DL feature extraction for the binary disease classification in medical imaging (The supplementary material, S6, provides the pseudocode outlining the study’s pipeline and workflow). The main findings demonstrate that carefully selected pre-processing steps, paired with appropriate, stable DL feature extractors, yield substantial and statistically significant gains in the mean sensitivity across histopathological (H&E-stained), radiological (chest X-ray), and ophthalmological (retina OCT) datasets. Critically, no single pre-processing configuration, including adjustment methods, filtering techniques, normalization schemes, or their combinations, was universally superior; optimal pipelines proved modality specific. Nevertheless, of the various pre-processing configurations that were examined, normalization was determined to be the most significant factor in improving the model’s efficiency. Despite the absence of a universally applicable normalization standard that would function perfectly in every scenario, normalization turned out to be indispensable, particularly in the context of employing DL architectures within the processing workflow. The architecture of CNNs is inherently tuned to the statistical distribution and scale of the training data. Consequently, bypassing the normalization stage can lead to significant performance declines. Without standardized scaling, random intensity values may distort feature maps, forcing the network to focus on noise rather than meaningful patterns. Conversely, implementing robust normalization ensures an even contribution from all input features, thereby reinforcing both model stability and its capacity to generalize to unseen data. Notably, the present study identified a distinctive divergence with respect to ResNet50, when deployed as the feature extractor. In this particular instance, the utilization of data that had not undergone additional normalization processes resulted in the mean sensitivity outcomes that were demonstrably superior. This outcome is presumably attributable to the fact that ResNet50, as a pre-trained DL model, was initially optimized and trained under specific data conditions that more closely aligned with our raw input. While it is imperative that adjustment and filtering methods be customized to suit the characteristics of a given dataset, normalization remains an essential element in the construction of an effective image analysis pipeline.

Among the five pre-trained DL architectures tested, VGG16 and DenseNet121 consistently delivered the most robust performance regardless of the preceding pre-processing choices, while average pooling generally outperformed max pooling.

These modality-dependent outcomes can potentially arise from the distinct physical and visual properties inherent to each imaging technique. Specifically, H&E-stained patches rely on colorimetric contrast between cellular structures; chest X-rays depend on differential photon absorption; and OCT scans utilize interferometric reflectance from retina layers. Consequently, adjustments to brightness or contrast, spatial filters (mean, median, or Gaussian), and normalization ranges interact differently with modality-specific noise, illumination gradients, and texture distributions. As demonstrated by false-color visualizations and boxplot summaries, a single pre-processing step may enhance diagnostic feature preservation in one modality while suppressing relevant details in another, explaining the absence of a universally transferable best practice.

Through an extensive evaluation of 248 distinct pre-processing combinations paired with five different DL feature extractors (yielding 1240 pipelines per dataset), this study conclusively closes the research gap described in the introduction. Previously, it was unclear whether model performance was primarily driven by the underlying architecture or by the specific pre-processing pipeline and feature extraction approach. The present findings demonstrate that improvements do not arise exclusively from either the feature extraction architecture or the pre-processing strategies. Instead, optimal performance in diagnostic tasks depends on effectively combining both aspects. Specifically, the results indicate that incorporating at least one well-suited pre-processing step alongside a robust DL feature extractor promotes a complementary interaction between preparatory transformations and the stability of the extractor, ultimately leading to the improved outcomes.

As observed, the superior consistency and robustness of VGG16 and DenseNet121 are evidenced by their emphasis on hierarchical, reusable features. In contrast, the greater variability of ResNet50 and InceptionV3 reveals the sensitivity of residual and multi-scale designs to pre-processing within the linear PCA-LDA classification framework. By demonstrating that even minimal, well-chosen pre-processing combined with a robust DL extractor reliably elevates classification efficacy, this study establishes an evidence-based rationale for moving beyond convention-driven pipelines.

However, several limitations inherent to the study design should be acknowledged. Although the three publicly available datasets were standardized to 224 × 224 pixels with balanced classes to enable a fair cross-modality comparison, they do not fully capture real-world variability encountered in clinical practice. This includes differences in H&E staining protocols between laboratories, variations in patient positioning and chest X-ray exposure settings, device-specific artifacts, and patient motion in OCT scans. Consequently, the performance gains and modality-specific optima reported here may not directly translate to diverse clinical data. Furthermore, while the balanced class distribution used in this study is necessary for a controlled comparison, it does not reflect the class imbalance commonly observed in real clinical datasets, in which pathological cases are often scarce. Therefore, future work should evaluate the robustness of the identified optimal pipelines under imbalanced conditions, as pre-processing and feature extraction strategies may interact differently with imbalanced data.

We extensively investigated the most commonly used pre-processing methods, trying to identify approaches applicable across a broad range of imaging modalities, with the goal of enhancing the generalizability of our findings. While other image enhancement techniques certainly exist, they were beyond the scope of this study. For feature extraction, we also employed five pre-trained DL networks with differing structures and architectures. We recognize that the exclusion of newer architectures or custom-trained models may constrain our conclusions. For example, more recent architectures, such as Vision Transformers (ViTs) and their variants, were not included in the present evaluation. These architectures have self-attention mechanisms that capture long-range spatial dependencies, which differ fundamentally from the local feature hierarchies learned by CNNs. Their behavior under varying pre-processing conditions may also differ substantially from the patterns observed here. This would be a significant area for future study. While testing all possible methods was impractical, this study offers a strong foundation for future research to build on with broader approaches.

Additionally, we restricted the classifier to a simple PCA-LDA model and the analysis to the binary tasks only, an assumption made to isolate the effects of pre-processing and feature extraction without confounding influences from complex hyperparameters or multi-class scenarios. This choice limits the generalizability of the findings to more advanced classifiers or multi-class diagnostic problems.

Finally, we performed the study using high-performance hardware GPU, to accelerate the computationally intensive calculations. While this enabled efficient processing and faster results, it also introduces a limitation regarding the scalability and applicability of the proposed pipeline in resource-constrained environments.

The present study demonstrated that tailored pre-processing coupled with a stable DL feature extraction approach is crucial for maximizing the model performance. However, translating these highly optimized, context-dependent findings into real-world diagnostic workflows presents significant challenges. This gap is not just theoretical; it is evidenced by specific limitations in clinical practice. Understanding these challenges and limitations can help guide future research by building upon the findings of this study. By prioritizing key parameters, objectives, and goals, subsequent studies can address these limitations and further advance the field.

## Conclusion

This study provides a systematic evaluation of how image pre-processing and DL feature extraction jointly influence the binary classification performance. The analysis spans three distinct medical imaging modalities: radiology (chest X-rays), pathology (H&E-stained colorectal tissue), and ophthalmology (retina OCT). We conducted a comprehensive assessment of configurations that included brightness and contrast adjustments, histogram equalization, and spatial filtering (mean, median, and Gaussian) using various kernel sizes and sigma values. These pre-processing steps were systematically combined with three normalization schemes, [min, max], [− 1, 1], and maximum value, as well as five benchmark DL architectures: VGG16, ResNet50, DenseNet121, MobileNetV2, and InceptionV3 for feature extraction. Finally, we utilized PCA-LDA classification and quantified the resulting impact using mean sensitivity as the primary metric.

The central finding is clear: no single pre-processing method or universal pipeline consistently outperformed the others. Optimal configurations were modality-specific, confirming the absence of a “one-size-fits-all” solution and underscoring the necessity of case-oriented optimization. Nevertheless, pre-processing remained indispensable. Skipping all steps consistently degraded performance, while even a single well-chosen operation notably improved the results. By systematically tuning pre-processing strategies alongside the choice of DL feature extractor, mean sensitivity rose substantially: from 74·9% to 96·95% (H&E), from 89·9% to 96·65% (chest X-ray), and from 82·4% to 98·8% (OCT). These improvements translate directly to fewer missed diagnoses, earlier intervention, and reduced clinician burden.

Among the explored DL feature extractors, VGG16 and DenseNet121 (closely followed by MobileNetV2) proved the most robust across all datasets, delivering the highest median sensitivities and lowest variability. In contrast, ResNet50 and InceptionV3 were more sensitive to pipeline choices, which highlights the critical interplay between architectural stability and pre-processing.

Our observations highlight that pre-processing and extracting key embedded features from medical image data is not only a technical procedure, but also a critical component in developing safe and effective AI diagnostic tools. Careful optimization of these processes is essential prior to real-world clinical deployment.

While our comprehensive evaluation demonstrates the effectiveness of context-specific pipelines, we acknowledge several important limitations. The three public datasets, though balanced and standardized to 224 × 224 pixels, may not reflect real-world clinical variability. Furthermore, the analysis was restricted to five pre-trained CNNs and basic PCA-LDA, limited to the binary classification tasks, and relied on high-performance GPUs; meanwhile, inter-institutional hardware and protocol differences may hinder immediate clinical translation. The development of a more reliable, generalizable, and deployable AI-driven diagnostic system will require the evolution of this context-dependent optimization into a multi-center validation on large-scale heterogeneous data using advanced classifiers and the exploration of multi-class scenarios.

## Supplementary Information

Below is the link to the electronic supplementary material.

Supplementary file1 (DOCX 5265 kb)

## Data Availability

The datasets analyzed during the current study are publicly available. The H&E-stained colorectal cancer and healthy tissue dataset is available from Zenodo at 10.5281/zenodo.1214456. The chest X-ray and retinal OCT datasets are available from Mendeley Data at 10.17632/rscbjbr9sj.2.
