# Supplementary material for: Systematic investigation of pre-processing and feature extraction techniques in medical image analysis
Source: Discov Artif Intell. 2026 Jul 22;6(1):700. doi: 10.1007/s44163-026-01768-1 (PMC13395947; doi:10.1007/s44163-026-01768-1)
Supplement: Supplementary file 1 — Supplementary file1 (DOCX 5265 kb) [file 44163_2026_1768_MOESM1_ESM.docx]

**Systematic Investigation of Pre-Processing and Feature Extraction Techniques in Medical Image Analysis**

**Pegah Dehbozorgi ^1,2^, Oleg Ryabchykov ^1,2^, Thomas W. Bocklitz ^1,2^**

^1^ Leibniz Institute of Photonic Technology, Member of Leibniz Health Technologies, Member of the Leibniz Centre for Photonics in Infection Research (LPI), Albert‑Einstein‑Strasse 9, 07745 Jena, Germany.

^2^ Institute of Physical Chemistry (IPC) and Abbe Center of Photonics (ACP), Friedrich Schiller University Jena, Member of the Leibniz Centre for Photonics in Infection Research (LPI), Helmholtzweg 4, 07743 Jena, Germany.

Corresponding author: Thomas Bocklitz

Email: ([Thomas.bocklitz@uni-jena.de](mailto:Thomas.bocklitz@uni-jena.de))

Phone: +49 3641-9-48328

**S1. Adjustment**

To improve the quality of raw medical images, we have chosen three widely recognized methods that can be applied to almost all image modalities. These methods consist of brightness adjustment, contrast adjustment, and histogram equalization. Brightness adjustment is a simple yet effective method that involves changing the brightness of an image by adding or subtracting a constant value from each pixel. This leads to a lighter or darker image. It affects all pixels equally, ensuring that the change is uniform across the entire image and all channels. Contrast adjustment is another technique within the SDMs group that enhances feature visibility in an image by modifying the brightness differences between objects or regions within the image. This process makes details more distinguishable, proving particularly beneficial for tasks such as object detection and image classification. Histogram equalization has been selected as the third method for image enhancement. Histogram is a graphical representation that shows the distribution of intensity values in an image, highlighting the number of pixels at each intensity level. Histogram equalization works by redistributing the most frequent intensity values, effectively stretching the overall intensity range of the image. This method typically boosts the global contrast of images where the data is concentrated in a narrow range of intensities, allowing areas with lower local contrast to gain more distinction. By implementing these adjustment methods, we aimed to enhance the visual quality of the images, making them more explicit and detailed.

**S2. Filtering**

Filters are essential tools in image pre-processing, particularly for noise reduction. They are matrices or kernels that are convolved over images to alter their appearance or extract specific features. Filters function by altering the pixel values of an image based on the values of neighboring pixels. In this study, we employed three commonly used filtering techniques, mean, median, and Gaussian, to investigate their effects on the medical image analysis through the binary classification tasks. To conduct a more thorough examination, we utilized three kernel sizes for the mean and median filters, and three different sigma values for the Gaussian filter. This approach enabled us to evaluate both the impact of the filtering techniques and the influence of kernel size and sigma value choices on the final analysis outcomes.

Mean filtering is a simple, intuitive, and easy-to-implement method for smoothing images, which reduces the intensity variation between adjacent pixels. The basic concept of mean filtering is to replace each pixel value in an image with the mean value of its neighbors, including itself. This process eliminates pixel values that are unrepresentative of their surroundings. Mean filtering is typically implemented as a convolution filter, using a kernel defining the shape and size of the neighborhood sampled to calculate the mean. While a standard 3 × 3 square kernel is commonly used, we also employed larger kernels, such as 5 × 5 and 7 × 7, to enhance the smoothing effect and achieve more substantial noise reduction. Mean filtering is simple, fast, and efficient for noise reduction, making it ideal for real-time scenarios and rapid processing, but it also has notable drawbacks. One major issue is the potential for blurring or loss of fine details, as the uniform averaging process can smooth out sharp edges, textures, and important image features, especially in high-frequency areas. Additionally, mean filtering can introduce unrealistic values near sharp transitions, affecting key image features.

Median represents the middle data point, with half the data being smaller and half larger. This makes it a robust estimator for removing outliers from a dataset. Similar to the mean filter, the median filter examines each pixel in the image and evaluates its neighboring pixels to determine if it is representative of its surroundings. The operation of the median filter is quite simple and can be summed up in three steps: neighborhood definition, sorting, and replacement. Simply it works by moving through the image one pixel at a time and replacing each pixel value with the median value of its neighboring pixels. This is done using a window or kernel that slides over the entire image, pixel by pixel. To find the median value, all the pixel values within the window are sorted in numerical order, and the median value is then used to replace the original pixel value. Since the selection of kernel size is vital in the process of median filtering, similar to mean filtering, kernels such as 3 × 3, 5 × 5, and 7 × 7 have been considered for applying median filtering. One of the primary advantages of the median filter over the mean filter is its ability to preserve edges and fine details. While the mean filter tends to blur edges and contours when noise is present, the median filter effectively suppresses noise while maintaining the sharpness of image boundaries. This feature makes the median filter valuable in applications where it is essential to preserve important structural information. However, the computational complexity of median filtering increases with larger neighborhood/kernel sizes, making it less suitable for real-time applications or processing large images.

Gaussian filtering is a commonly used technique in image pre-processing for smoothing and noise reduction. The filter is named after the Gaussian distribution, also known as the normal distribution, which represents the bell-shaped curve that describes the probability distribution of a continuous random variable. When applied to an image, the Gaussian filter works as a weighted averaging mechanism. It assigns higher weights to pixels closer to the center, and gradually lower weights to those farther away, following the Gaussian distribution. Consequently, each pixel in the image is replaced with a weighted average of its neighboring pixels, with the weights determined by the Gaussian kernel. While Gaussian filtering is effective for smoothing and noise reduction, it can also blur sharp edges and fine details due to the influence of neighboring pixels in the smoothing process, which ultimately diminishes overall image sharpness. This over-smoothing may obscure important textures in high-frequency areas. Furthermore, the convolution process can be computationally intensive, particularly for larger images and higher sigma values, resulting in longer processing times. Pros and cons of each filtering method encouraged us to investigate them systematically and comprehensively, to carefully examine their associated and potential effects on the image analysis.

**S3. Normalization**

ML algorithms typically assume that all features contribute equally to the final prediction. However, this assumption can fail when features vary in range and unit, affecting their perceived importance. Normalization addresses this issue by standardizing the numerical values of features, ensuring uniformity. When all features in a dataset are on the same scale, it becomes easier to identify and visualize relationships between different features and make meaningful comparisons. Normalization is especially beneficial for algorithms that rely on distance metrics, as it prevents features with larger scales from dominating the learning process. In this study, we considered three different normalization ranges. The first range explored is the commonly used min-max range, which transforms features to a specified range, typically between 0 and 1. The second range examined is [-1, 1], offering a different normalization perspective. Additionally, we investigated the effect of normalizing pixel values by dividing them by their maximum value (norm to max). These normalization techniques were applied to standardize pixel values across all images, ensuring fair comparisons and consistent data pre-processing.

**S4. Pre-processing combinations**

Our pre-processing pipeline applies three sequential stages: adjustment, filtering, and normalization, across different combinations of the methods. This is followed by feature extraction using two pooling modes: average and max. The total of 248 pipelines arise from 124 unique pre-processing combinations (adjustment × filtering × normalization), each of which is tested under two pooling modes (124 × 2 = 248). The 124 pre-processing combinations are derived from three unique cases based on whether adjustment and filtering are applied. Case A involves active adjustment with active filtering and has 108 pre-processing combinations. Each of the three adjustment methods (brightness, contrast, and histogram equalization) is combined with all the incorporated filtering configurations. Filtering comprises three methods, each with its own set of parameters: Mean filtering with three kernel sizes (3 × 3, 5 × 5, and 7 × 7); median filtering with three kernel sizes (3 × 3, 5 × 5, and 7 × 7); and Gaussian filtering with three sigma values (σ = 0.5, 1, and 2). This yields nine filtering configurations in total. Note that kernel size is the relevant parameter for mean and median filters, while sigma is the relevant parameter for Gaussian filtering. These parameters are not interchangeable and cannot be combined. This gives: Three adjustments × nine filtering options × four normalization schemes = 108 pre-processing combinations. Case B: Active adjustment without filtering (12 pre-processing combinations): Each of the three adjustment methods is evaluated independently, without any filtering step applied. This tests the contribution of the adjustment method alone, independent of filtering. This gives: Three adjustments × one filter × four normalization schemes = 12 pre-processing combinations. Case C: No adjustment or filtering; pure baseline (four pre-processing combinations): This condition serves as the minimal pre-processing baseline, showcasing the contribution of normalization alone. This gives: 1 (no adjustment) × 1 (no filtering) × 4 normalization schemes = 4 pre-processing combinations. Therefore, the total number of unique pre-processing combinations is: 108 + 12 + 4 = 124. The total number of pipelines, including both pooling modes, is 248 (124 × 2).

**S5. Analysis results: Image pre-processing effect: Chest X-ray and retina OCT datasets**

Fig. S1(a) illustrates that applying contrast adjustment led to the higher mean sensitivity values in the chest X-ray analysis. Consistent with the observations from the H&E-stained dataset, Fig. S1(b) shows that the absence of a dominant color in the normalization process suggests that no specific normalization range consistently enhances the classification performance. However, it is notable that images without normalization exhibited a significant decrease in the mean sensitivity values. Fig. S1(c) explores the effects of various filtering techniques, demonstrating that it is challenging to identify a consistently best-performing method. Nevertheless, as shown in Fig. S1(d), employing the average pooling mode for feature extraction continues to enhance the performance of the classification models. By experimenting with different configurations, we enhanced the classifier's mean sensitivity from 89·9%, obtained through histogram equalization, a (7 × 7) median filtering kernel, and max pooling without normalization, to 96·3%. This improvement was achieved by implementing contrast adjustment, eliminating filtering, normalizing to [-1, 1], and employing the average pooling for feature extraction.

A similar trend observed in the H&E-stained and chest X-ray datasets was also evident in the retina OCT dataset. Fig. S2 indicates that no single pre-processing technique or combination of techniques consistently enhances the model's performance, as reflected in the mean sensitivity values. In this instance, the classification score improved from 82·95%, achieved using a combination of contrast adjustment, median filtering (with a kernel size of 7 × 7), and no normalization with max pooling, to 98·45% by applying no adjustments, no filtering, normalizing the images to the [-1, 1] range, and extracting key features using average pooling.


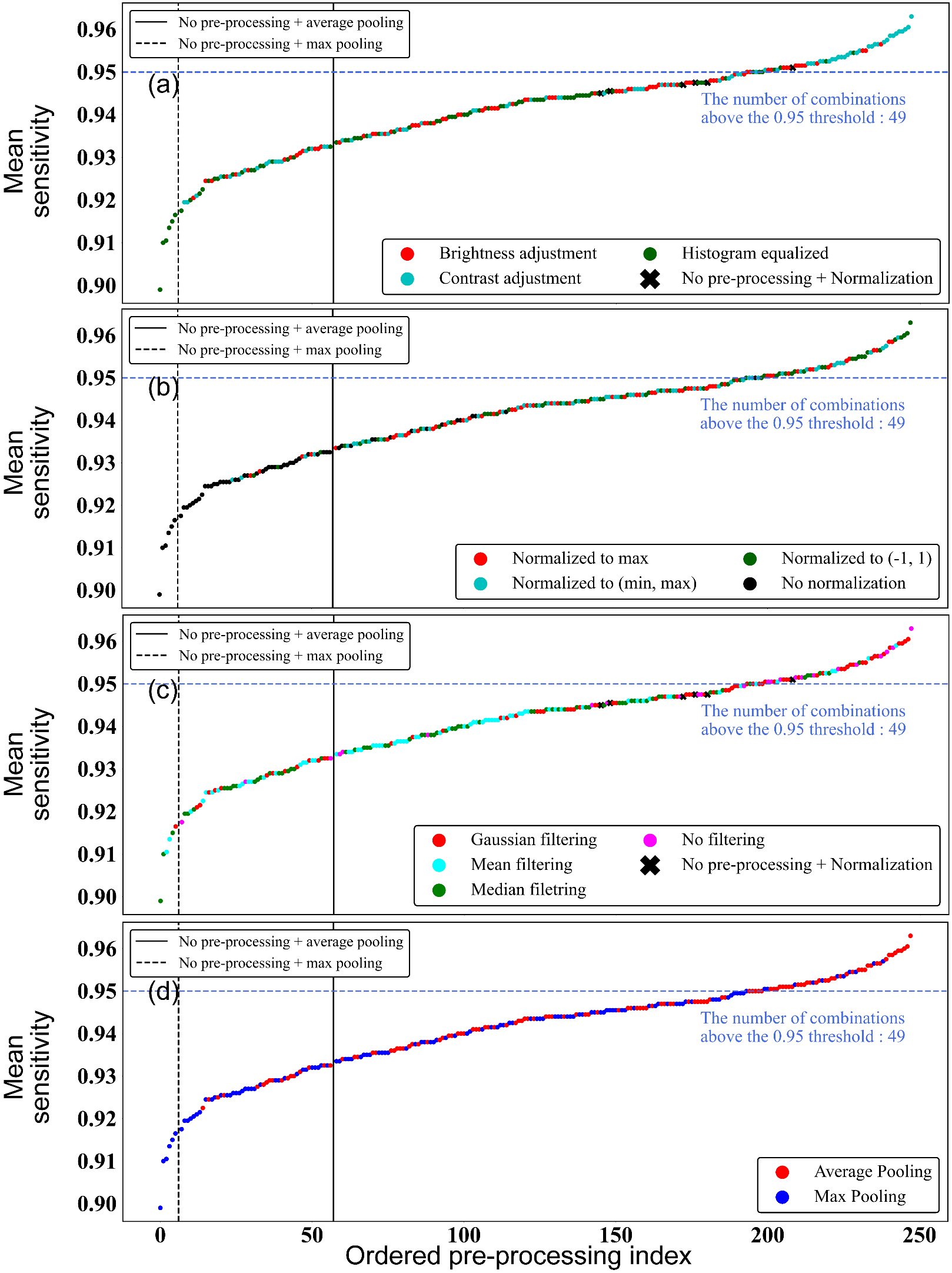


**Fig. S1** Optimization of the classification models for the chest X-ray dataset using DenseNet121 as the feature extractor. This figure illustrates the effects of various pre-processing techniques on the classification models (248 distinct models). Specifically, it examines different adjustment methods, filtering techniques with varying kernel sizes and sigma values, and the application of different normalization ranges to generate the pre-processed images as the input for the binary classification models. (a) No single adjustment technique consistently contributes to higher mean sensitivity values, as evidenced by the absence of a dominant color across the employed adjustment methods. However, when compared to the other implemented enhancement techniques, contrast adjustment contributed slightly to the best-performing models, delivering higher mean sensitivity values overall. (b) The analysis presents a comparative evaluation of different normalization ranges. While no single normalization approach emerged as universally effective in improving model performance, cases without normalization generally resulted in lower mean sensitivity values, suggesting that applying some form of normalization is beneficial in most scenarios. (c) The figure also illustrates the lack of a consistent color pattern among the implemented filtering techniques, reinforcing the idea that there is no one-size-fits-all solution for filtering. This variability suggests that filtering methods should be chosen based on the specific characteristics of the dataset, as well as the levels of noise, artifacts, and measurement errors present. (d) Finally, considering the pooling mode, the reddish color mainly observed around the higher mean sensitivity values suggests the effectiveness of average pooling on model performance. However, the overall mixture of blue and red colors makes it challenging to draw a robust conclusion. The dashed blue lines in (a), (b), (c), and (d) represent the established threshold (around 95%), with 49 classification models exceeding it, showcasing the effectiveness of these models for medical images and diagnostic tasks


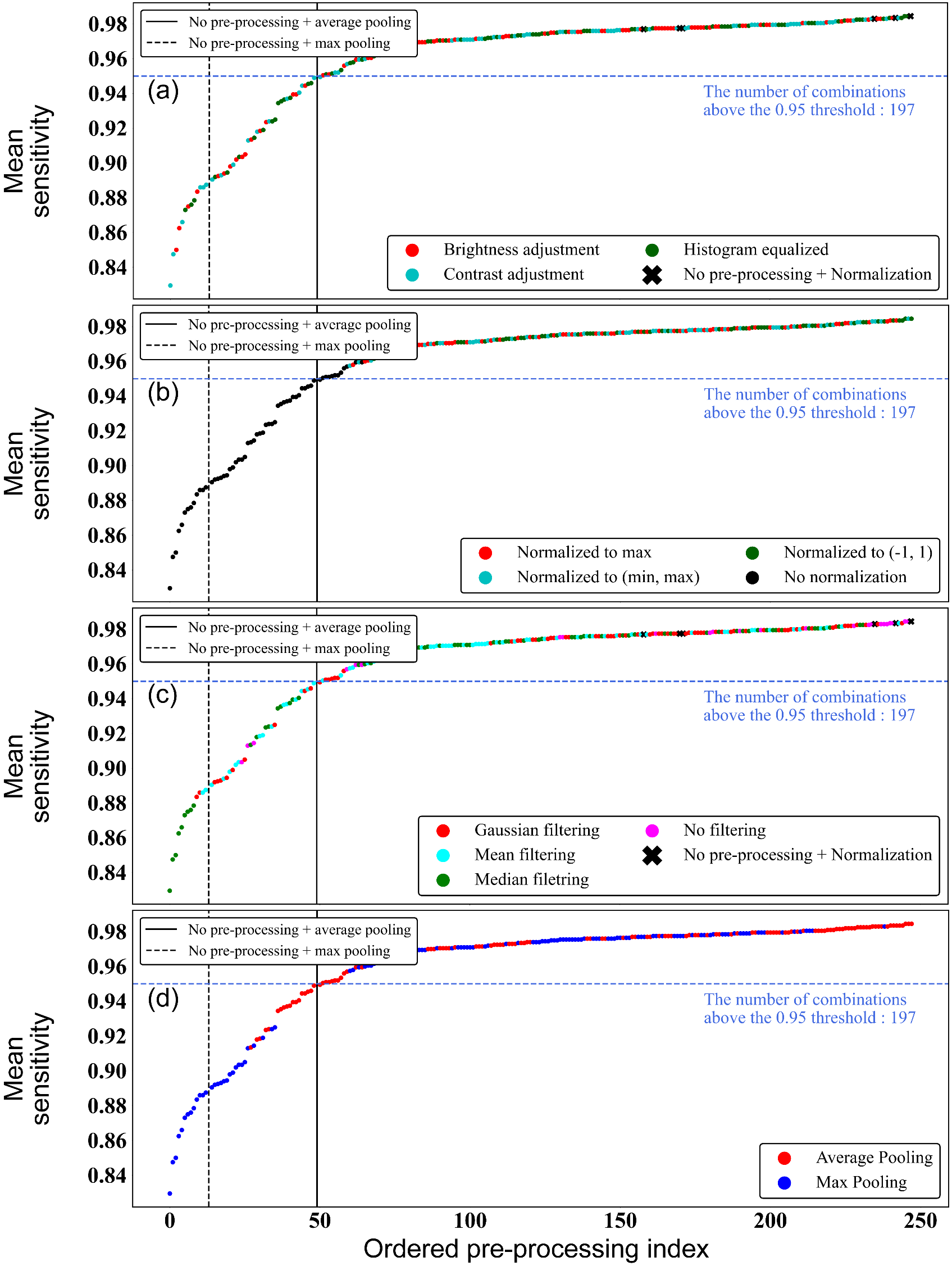


**Fig. S2** Optimization of the classification models for the retina OCT dataset using DenseNet121 as the feature extractor. This figure illustrates the effects of various pre-processing techniques on the classification models (248 distinct models). Specifically, it examines different adjustment methods, filtering techniques with varying kernel sizes and sigma values, and the application of different normalization ranges to generate the pre-processed images as the input for the binary classification models. (a) No single adjustment technique consistently enhances mean sensitivity values, as indicated by the lack of a dominant color across the various adjustment methods employed. (b) The analysis offers a comparative assessment of various normalization ranges. Although no single normalization method proved to be universally superior in raising model performance, the absence of normalization often led to reduced mean sensitivity values, indicating that incorporating normalization is advantageous in most cases. (c) This figure provides additional insights into the implications of different filtering methods. The mixture of colors and lack of a consistent pattern indicate that no single filtering method consistently yields high accuracy. Instead, various techniques need to be tested, and the most suitable one should be selected based on the specific characteristics of the data at hand. (d) When examining the pooling mode, the predominantly red coloration around the regions of higher mean sensitivity value highlights the potential effectiveness of average pooling in enhancing model performance. Nevertheless, the broader distribution of both blue and red colors across the spectrum introduces variability, making it difficult to draw definitive conclusions about the superiority of any single pooling method. The dashed blue lines in (a), (b), (c), and (d) represent the established threshold (around 95%), with 197 classification models exceeding it, showcasing the effectiveness of these models for medical images and diagnostic tasks

**TABLE S1** Performance metrics for the binary classification tasks using the H&E-stained dataset and DenseNet121 as the employed feature extractor. This table presents the evaluation metrics for the optimal, suboptimal and two baseline configurations

| Model configuration | Sensitivity | Specificity | Precision | Mean sensitivity | Mean specificity | Mean precision | F1 score | Accuracy |
| --- | --- | --- | --- | --- | --- | --- | --- | --- |
| Optimal (Fig. 6) | 0.965 | 0.967 | 0.967 | 0.966 | 0.966 | 0.966 | 0.9659 | 0.966 |
| Suboptimal (Fig. 6) | 0.854 | 0.89 | 0.885 | 0.872 | 0.872 | 0.8724 | 0.8719 | 0.872 |
| Baseline: No pre-processing + average pooling (Fig. 6) | 0.906 | 0.942 | 0.9398 | 0.924 | 0.924 | 0.9245 | 0.9239 | 0.924 |
| Baseline: No pre-processing + max pooling (Fig. 6) | 0.873 | 0.922 | 0.9179 | 0.8975 | 0.8975 | 0.8984 | 0.8974 | 0.8975 |

**TABLE S2** Performance metrics for the binary classification tasks using the chest X-ray dataset and DenseNet121 as the employed feature extractor. This table presents the evaluation metrics for the optimal, suboptimal and two baseline configurations

| Model configuration | Sensitivity | Specificity | Precision | Mean sensitivity | Mean specificity | Mean precision | F1 score | Accuracy |
| --- | --- | --- | --- | --- | --- | --- | --- | --- |
| Optimal (Fig. S1) | 0.949 | 0.977 | 0.9763 | 0.963 | 0.963 | 0.9633 | 0.9629 | 0.963 |
| Suboptimal (Fig. S1) | 0.865 | 0.933 | 0.9281 | 0.899 | 0.899 | 0.9008 | 0.8988 | 0.899 |
| Baseline: No pre-processing + average pooling (Fig. S1) | 0.923 | 0.948 | 0.9466 | 0.9355 | 0.9355 | 0.9357 | 0.9354 | 0.9355 |
| Baseline: No pre-processing + max pooling (Fig. S1) | 0.912 | 0.958 | 0.9559 | 0.935 | 0.935 | 0.9359 | 0.9349 | 0.935 |

**TABLE S3** Performance metrics for the binary classification tasks using the retina OCT dataset and DenseNet121 as the employed feature extractor. This table presents the evaluation metrics for the optimal, suboptimal and two baseline configurations

| Model configuration | Sensitivity | Specificity | Precision | Mean sensitivity | Mean specificity | Mean precision | F1 score | Accuracy |
| --- | --- | --- | --- | --- | --- | --- | --- | --- |
| Optimal (Fig. S2) | 0.994 | 0.975 | 0.9754 | 0.9845 | 0.975 | 0.984 | 0.9844 | 0.9845 |
| Suboptimal (Fig. S2) | 0.806 | 0.853 | 0.8457 | 0.8295 | 0.8295 | 0.8302 | 0.8294 | 0.8295 |
| Baseline: No pre-processing + average pooling (Fig. S2) | 0.956 | 0.942 | 0.9428 | 0.949 | 0.949 | 0.9490 | 0.9489 | 0.949 |
| Baseline: No pre-processing + max pooling (Fig. S2) | 0.892 | 0.885 | 0.8857 | 0.8885 | 0.8885 | 0.8885 | 0.8884 | 0.8885 |

**TABLE S4** Feature extraction time and memory usage per model for the H&E-stained dataset, reported for a single pipeline combination using raw, unprocessed images

| Feature extraction model | Time for extraction (For one single combination (mins)) | Memory usage (MB) |
| --- | --- | --- |
| VGG16 | 11.9 | 20.2 |
| ResNet50 | 14 | 88.3 |
| InceptionV3 | 14.4 | 93.7 |
| DenseNet121 | 14.9 | 46.9 |
| MobileNetV2 | 10.4 | 51.3 |

**TABLE S5** Feature extraction time and memory usage per model for the chest X-ray dataset, reported for a single pipeline combination using raw, unprocessed images

| Feature extraction model | Time for extraction (For one single combination (mins)) | Memory usage (MB) |
| --- | --- | --- |
| VGG16 | 11.24 | 23.4 |
| ResNet50 | 13.82 | 93.7 |
| InceptionV3 | 13.9 | 93.7 |
| DenseNet121 | 10.74 | 46.9 |
| MobileNetV2 | 10.14 | 50.6 |

**TABLE S6** Feature extraction time and memory usage per model for the retina OCT dataset, reported for a single pipeline combination using raw, unprocessed images

| Feature extraction model | Time for extraction (For one single combination (mins)) | Memory usage (MB) |
| --- | --- | --- |
| VGG16 | 10.9 | 23.4 |
| ResNet50 | 13.42 | 93.7 |
| InceptionV3 | 13.68 | 93.7 |
| DenseNet121 | 14.53 | 46.9 |
| MobileNetV2 | 10.2 | 50.6 |

**S6. Pseudocode for the presented analysis:**

INPUT

Datasets: D_HE (H&E-stained dataset), D_XR (Chest X-ray dataset), D_OCT (Retina OCT dataset)

- train/test already split: 10000 train/ 2000 test per dataset
- balanced classes, all images resized to 224 × 224 px

STEP 1: DEFINE PRE-PROCESSING OPTIONS

Adjustments = {brightness, contrast, histogram equalization, none}

Filters = {mean, median, Gaussian, none}

Kernels = {3 × 3, 5 × 5, 7 × 7}

Sigmas = {0.5, 1, 2}            ## Gaussian only

Normalizations = {[min, max], [−1, 1], max scaling, none}

## Build all valid combinations (fixed order: adjust → filter → normalize)

Combinations = []

FOR each adjustment:

FOR each filter + kernel/sigma:

FOR each normalization:

Combinations.add (adjustment, filter, kernel/sigma, normalization)

FOR each adjustment (no filter):

FOR each normalization:

Combinations.add (adjustment, none, normalization)

Combinations.add (none, none, each normalization)

STEP 2: LOAD FEATURE EXTRACTORS

Pre-trained DL models = {VGG16, ResNet50, DenseNet121, MobileNetV2, InceptionV3}

- loaded with ImageNet weights, frozen (no fine-tuning)

pooling modes = {average pooling, max pooling}

STEP 3: RUN ALL PIPELINES

Results = []

FOR each dataset (D_HE, D_XR, D_OCT):

FOR each combination (adjustment, filter, kernel/sigma, normalization)

FOR each pooling mode (average pooling, max pooling):

FOR each model (VGG16, ResNet50, DenseNet121, MobileNetV2, InceptionV3):

## Pre-process

train_images = apply (train set, adjustment, filter, kernel/sigma, normalization)

test_images = apply (test set, adjustment, filter, kernel/sigma, normalization)

## Feature extraction

train_features = model.extract(train_images, pooling)

test_features = model.extract(test_images, pooling)

## Classify with PCA-LDA sweep PCA components 1→ 100

best_sensitivity = 0

FOR n = 1 TO 100:

reduced_train = PCA(n). fit_transform( train_features )

         reduced_test = PCA(n). transform(test_features)

         predictions   = LDA (). fit(reduced_train). predict (reduced_test)

         sensitivity   = mean_sensitivity(test_labels, predictions)

         IF sensitivity > best_sensitivity → best_sensitivity = sensitivity

## Store results

results. save (dataset, combination, pooling, model, best_sensitivity)


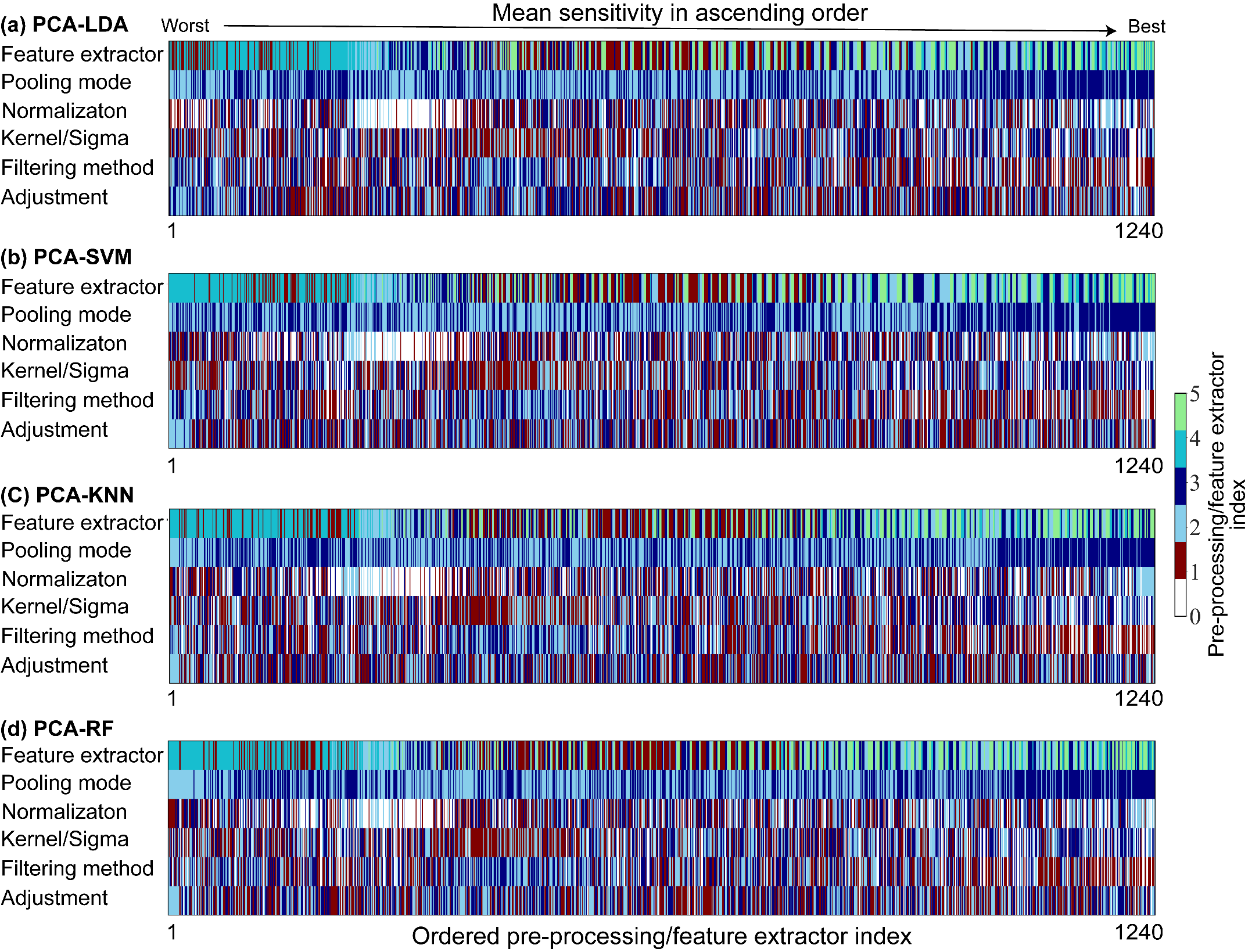


**Fig. S3** Impact of the pre-processing and feature extraction strategies across alternative classifiers on the H&E-stained dataset. In order to assess the robustness of the findings obtained with the (a) PCA-LDA framework on the more complex H&E-stained dataset, three alternative classifiers were evaluated: The following classifiers were evaluated: (b) PCA-SVM, (c) PCA-KNN, and (d) PCA-RF. For each of the 1240 possible combinations of pre-processing parameters (pooling mode, normalization, kernel/sigma, filtering method, and adjustment) and feature extraction methods, the mean sensitivity values were ranked in ascending order for each classifier. The color of each column encodes the pre-processing and feature extractor index according to the shared color bar. In line with the preliminary observations made from the PCA-LDA model, the pre-processing exhibited a highly mixed color distribution across all the integrated classifiers, indicating the absence of a universally optimal pre-processing strategy for medical image analysis. Conversely, the feature extraction row demonstrated a certain degree of color consistency, thereby indicating that feature extraction tends to exert a more systematic and reproducible influence on the classification performance. These findings underscore the conclusion that, while no specific pre-processing configuration generalizes across diverse settings, the selection of feature extractor contributes more consistently to the model performance, irrespective of the classifier employed


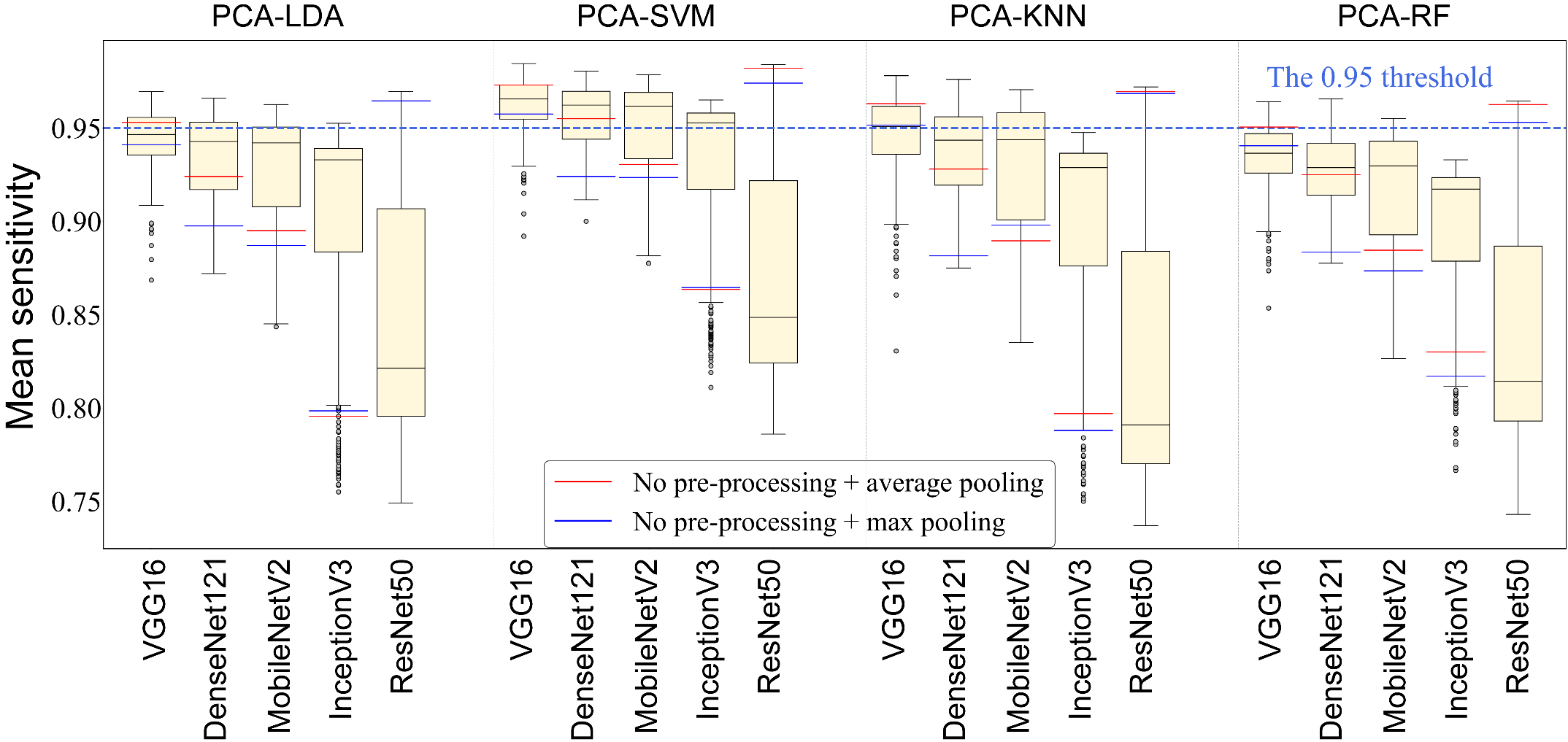


**Fig. S4** A comparative analysis of deep feature extractors using different PCA-based classifiers on the H&E-stained dataset. The boxplots illustrate the mean sensitivity achieved by deep features extracted from VGG16, DenseNet121, MobileNetV2, InceptionV3, and ResNet50 on the H&E-stained dataset when combined with four PCA-based classifiers: PCA-LDA, PCA-SVM, PCA-KNN, and PCA-RF. Across all the classifier models, a highly consistent performance pattern was observed, indicating that the relative ranking of the feature extractors remained largely unchanged irrespective of the downstream classifier. VGG16, DenseNet121, and MobileNetV2 have been shown to consistently yield high and stable mean sensitivity values. By contrast, InceptionV3 and ResNet50 have been observed to demonstrate comparatively lower performance and greater variability. It is interesting to note that the PCA-LDA classifier produced mean sensitivity values that were comparable to those obtained with more complex classifiers, including SVM, KNN, and Random Forest. This consistency suggests that the discriminative information captured by the extracted features plays a more influential role than the choice of classifier itself. The findings indicate that the selection of the classifier had a negligible effect on the observed performance trends, thereby underscoring the robustness and generalizability of the extracted feature representations across varied classification frameworks
